# A direct method for the identification of patterns of care using administrative databases: the case of breast cancer

**DOI:** 10.1007/s10198-021-01327-8

**Published:** 2021-07-26

**Authors:** Susanna Busco, Andrea Tavilla, Anna Gigli, Tania Lopez, Daniela Pierannunzio, Sandra Mallone, Stefano Guzzinati, Giulia Capodaglio, Francesco Giusti, Catia Angiolini, Silvia Francisci

**Affiliations:** 1 UOC Programmazione Controllo E Governo Clinico-ASL Latina, Latina, Italy; 2 National Centre for Disease Prevention and Health Promotion, National Institute of Health, Rome, Italy; 3 Institute for Research on Population and Social Policies, National Research Council, via Palestro 32, 00185 Rome, Italy; 4 Veneto Tumour Registry, Azienda Zero, Padua, Italy; 5 Regional Epidemiology Service, Azienda Zero, Padua, Italy; 6 Francesco Giusti, Brussels, Belgium; 7 Breast Oncology Unit, Careggi University Hospital, Florence, Italy

## Abstract

**Objectives:**

To identify and provide lists of procedures and drugs related to diagnosis and treatment of breast cancer. These lists can be used for the estimation of the cost of illness.

**Methods:**

The method consists of identifying lists of procedures/interventions/drugs related to the tumour of interest, drawn by a panel of expert clinicians and oncologists on the basis of clinical guidelines and current practice. The lists are applied to data referring to breast cancer female patients, collected by population-based Cancer Registries and linked at individual level with information on health care treatments. A comparison with lists obtained via the matched control method is implemented.

**Results:**

The distribution of administered procedures and drug prescriptions is coherent with the patient clinical pathway: surgery is the main cause of hospitalization in the first year since diagnosis, diagnostic and monitoring interventions are more frequent in the following years (recurrences detection), and at end-of-life (palliative care). Most outpatient services are due to diagnosis and monitoring, one third of services in the first year since diagnosis is radiotherapy and chemotherapy. Drugs prescribed to patients and sold in pharmacy include hormonal drugs as first course treatment and analgesics as palliative care.

**Conclusions:**

This direct method represents a valid alternative to the matched control method in describing patterns of care and costs related to the entire disease pathway. It is particularly suitable in case of cancer sites with complex patterns of care, such as breast cancer. The lists of codes developed here are based on international classification systems and can be easily applicable to other countries.

**Supplementary Information:**

The online version contains supplementary material available at 10.1007/s10198-021-01327-8.

## Introduction

Administrative databases are more and more often used for estimating patterns of care and cost profiles of chronic diseases, such as cancer. Cost-of-illness studies are challenging because of the difficulties in identifying those costs that are due specifically to an illness.

There are two broad approaches used in cost-of-illness studies: the total cost approach, which consists of adding up all health expenditures referred to a cohort of patients or a selection of costs directly related to the disease of interest; and the matched control approach, which uses a comparison group of patients to isolate the incremental cost due to the disease of interest [1–3]. The matched control approach requires a proper matching between cases and controls, with respect to all confounding factors. This *indirect* approach is typically used in studies on the cost of cancer in the United States, based on SEER and Medicare data [4, 5], the latter containing information on cancer as well as non-cancer patients, and in New Zealand based on administrative health care datasets [6]. The total cost approach, when restricted to expenditures directly related to the disease of interest, requires an accurate and complete identification of these expenditures. This *direct* approach, also known as *Attribution* method [2], has been applied to identify and compare patterns of treatment of colorectal cancer patients during the first year since diagnosis between Italy and the United States [7].

The choice between these two approaches depends on the scope of the analysis, on the type of information available and on the illness of interest, as it will be illustrated in the discussion.

Aim of this paper is to identify lists of diagnostic and intervention procedures and drugs related to breast cancer using international classification systems. The resulting lists are obtained applying the direct method and are compared with those obtained using the indirect approach, in a selected subset of the study cohort for which information before cancer diagnosis is available.

The methodology is based on cancer registry data linked with administrative data and requires a strong involvement of expert clinicians and oncologists. Lists have been applied to estimate costs due to breast cancer diagnosis, treatment and follow-up in eight Italian regions, within the Epicost study [8]. Epicost represents the first attempt in Italy to provide population-based estimates of direct cancer costs across the patient pathway, when data at individual level are available.

## Materials and methods

### Data sources

The study includes data from population-based Cancer Registries (CRs) linked to four administrative sources that provide information on the following health care services: hospitalizations (Hospital Discharge database-HD), ambulatory services (Outpatient Services database-OPS), drugs prescribed to a patient and sold by a pharmacy (Drug Prescriptions database-DP), and high cost drugs administered to a patient in hospital (Hospital Pharmacy database-HP).

Information is collected at individual level and includes an anonymous personal identification code used for the record linkage with the CRs database, to trace all services provided to a single patient in the cancer registry in a given period of time. All personal data concerning individuals involved in this study were managed according to the current Italian privacy legislation [9] which identifies cancer registries as collectors of personal data for surveillance purposes without any need for explicit individual consent. The descriptive analysis of individual data did not involve any direct or indirect intervention on the population considered.

Population-based CRs collect data on all cancer diagnoses occurring in the population resident in the area covered by cancer registration. The following variables are included: date of birth, date of diagnosis, gender, vital status, topography and morphology code (according to ICDO-3 classification), diagnostic confirmation.

HD contains information on hospitalization. Each record refers to a single hospital episode and includes demographic variables (date of birth, sex, place of birth, place of residence), clinical variables (main diagnosis and up to five secondary diagnoses, main intervention/procedure and up to five secondary interventions/procedures coded according to ICD9-CM classification [10]), administrative variables (coded according to the DRG coding system, dates of admission and discharge). In this work, 24,480 records were analysed.

OPS contains information on outpatient services (for example diagnostic tests, ambulatory procedures). Each record refers to a single outpatient episode and includes the following variables: type of procedure and date of the episode. Procedures are coded according to a national coding system, based on ICD9-CM classification; 593,393 records were analysed.

DP and HP contain information on drugs and include the following variables: type of drug, coded according to the Anatomical Therapeutic Chemical (ATC) classification system [11] and date of prescription. Each record refers to a single drug; 313,719 records were analysed.

### Study cohort

This study involves 8 Italian CRs, located in 8 out of 20 Italian regions, having at least 8 years of cancer registration: Veneto, Friuli Venezia Giulia, Milano in Northern Italy; Umbria, Firenze-Prato, Latina in Central Italy; Napoli, Palermo in Southern Italy. Regional coverage varies between 100% (Friuli Venezia Giulia and Umbria regions) and 10% (Latina in Lazio region); overall, they cover just over 10 million people, corresponding to about one sixth of the Italian population. A more detailed description of the study cohort can be found in [8].

The study design is cross-sectional: the study cohort includes patients alive at the most updated prevalence date available at the time of data collection, i.e., Jan 1st 2011, and having being diagnosed in a period of 8 years before (from Jan 1st 2003 to Dec 31st 2010). A total number of 49,270 prevalent women with breast cancer (ICD9-CM C50) are in the study cohort for the pool of CRs. Administrative data containing information on interventions, procedures and drugs is available for a 3-year interval, covering 2 years before to 1 year after prevalence date (from Jan 1st 2009 to Dec 31st 2011).

## Methods

We use a *direct method* to identify those procedures, interventions and drugs related to the tumour diagnosis, treatment and clinical follow-up. The method consists of identifying lists of *events*, i.e., procedures/interventions/drugs, related to the tumour of interest, drawn by a panel of expert clinicians and oncologists on the basis of clinical guidelines [12]. The lists are associated to ICD9-CM codes for the identification of hospital and outpatient events and ATC codes for the identification of drugs. An event is cancer-related if the associated code belongs to the cancer-related list. In the case of hospitalization, a single episode may be associated with up to six diagnoses and six procedures/interventions, and a hospitalization is considered cancer-related if at least one code belongs to the cancer-related list.

The lists of cancer-related codes (D-lists) are reported in Online Appendix 1 separately for each type of service: hospitalization, outpatient, drugs. Each code is assigned to a clinically relevant category and a sub-category defined according to the type of the intervention as for hospitalizations and outpatient services or the type of treatment as for drugs.

We compare the results obtained using the direct method with those obtained by applying the matched control method to a subset of patient: according to this method, a comparison group of subjects without cancer diagnosis is matched with cases for all possible confounders and used to estimate the incremental access to health care services due to the breast cancer diagnosis. In our data set, since information on events is available for 2 years before prevalence date, for those patients in initial phase (i.e., diagnosed during year 2010) we also have information in a period prior to diagnosis, when we can assume that they undertook the same procedures as the general non-cancer population. Thus, the same patients in the initial phase are used as *self-controls* in the time interval spanning from 2 months up to 12 months before diagnosis [13]. Each patient belonging to this subset of the study cohort is linked to all events in the 10 months post**-** (cases) and in the 10 months pre- (controls) cancer diagnosis. Notice that 2 months immediately prior to diagnosis were left out, to avoid possible biases due to cancer-related investigations in the pre-diagnostic period. The lists of cancer-related codes identified for hospitalizations, outpatient services and drugs by the matched control method (C-lists) are obtained in the following way: for each code, identifying a procedure/intervention/drug, cases and controls average occurrences are compared using a two-sample t-test for independent samples with unequal variances (Satterthwaite’s Method) [14] (see Online Appendix 3). Whenever the difference between cases and controls average occurrences is statistically significant (*p* < 0.05), the corresponding code is included. To measure the concordance between the C-lists and the D-lists we compute the ratio between the number of codes included in both C-lists and D-lists and the number of codes included in the D-lists. Since frequencies of single codes can vary greatly, each code is weighted according to its number of occurrences. The cancer-related codes are then applied to the entire study cohort and distribution of occurrences are obtained for each phase of care, thus providing an overview of the patterns of care. To this purpose, we define three mutually exclusive phases of care: initial year following diagnosis (initial phase), last year of life (final phase), and the time between initial and final phase (continuing phase) and assign each case in the cohort to the phase of care she belongs on *prevalence date*, in the following way: if she had been diagnosed within 12 months before prevalence date and she is alive 12 months after prevalence date, she belongs to the *initial phase*; if she had been diagnosed more than 12 months before prevalence date and she is alive 12 months after prevalence date, she belongs to the *continuing phase*; if she died within 12 months after prevalence date, regardless of when she had been diagnosed, she belongs to the *final phase*. Women dying for causes other than cancer are considered as censored. It should be noticed that, although during her life span each woman can contribute to more than one phase of care, on prevalence date each patient belongs to only one phase of care, that is the phases of care are mutually exclusive. For each patient in the cohort we analyse information on events occurring in a 12-month period. This period spans from diagnosis onwards for patients in the initial phase, from death backward for patients in the final phase and from 6 months before to 6 months after the prevalence date for patients in the continuing phase.

## Results

The direct method identifies 202 codes for hospitalization, 250 codes for outpatient procedures and 60 codes for pharmaceutical prescriptions. Codes are listed in Online Appendix 1. Of these, 99.6% codes for hospitalizations, 97.6% for outpatient services and 99.9% for drugs have been confirmed using the matched control method. The lists are applied to the study cohort, which is made of 7501 patients in the initial phase, 39,369 in the continuing phase and 2400 in the final one, corresponding to 15, 85 and 5% of the cohort in initial, continuing and final phase, respectively.

Tables 1–3 describe the distribution by phase of care of the D-lists codes occurrences (grouped into categories and sub-categories), each table describes a different health care service. Tables A2.1–A2.3 in Online Appendix 2 report, separately for each phase of care, the ten most frequently used codes in each D-list. Notice that at the time of the study information collected in the HP database, containing high cost drugs administered in hospital, such as some types of chemo- and immuno-therapeutic drugs, was incomplete and very variable among regions. Consequently, results regarding the HP database are merely qualitative: the ATC codes were subjected to validation and are listed in Online Appendix 1, but no frequency table is shown.

**Table 1:** Distribution of hospital discharges by category, sub-category and phase of care: absolute values (N) and percent distribution (%) within each phase of care

		Initial		Continuing		Final	
		*N*	%	*N*	%	*N*	%
Category	Sub-category						
Surgery		8021	67.6	2560	28.8	93	2.5
	Lumpectomy	4931	41.6	505	5.7	30	0.8
	Surgery	1826	15.4	260	2.9	31	0.8
	Lymphadenectomy	743	6.3	128	1.4	16	0.4
	Plastic surgery	433	3.6	1646	18.5	15	0.4
	Radical and reconstructive surgery	88	0.7	21	0.2	1	0.0
Chemotherapy		2150	18.1	1668	18.7	900	24.2
Diagnosis and monitoring		1397	11.8	4045	45.4	2,195	59.1
	Biopsy	356	3.0	342	3.8	49	1.3
	Cardiologic assessment	340	2.9	1019	11.4	446	12.0
	Diagnosis	269	2.3	961	10.8	541	14.6
	High diagnostic	231	1.9	807	9.1	569	15.3
	Conventional radiology	101	0.9	580	6.5	359	9.7
	Ultrasonography	85	0.7	270	3.0	120	3.2
	Invasive procedure	8	0.1	42	0.5	100	2.7
	Other diagnostic procedure	7	0.1	30	0.3	11	0.3
Radiotherapy		173	1.5	108	1.2	101	2.7
Support therapy		78	0.7	319	3.6	348	9.4
Biologic therapy		35	0.3	102	1.1	19	0.5
Transfusion		12	0.1	98	1.1	58	1.6
Total		11,866	100.0	8900	100.0	3714	100.0

**Table 2:** Distribution of Outpatient procedures by category, sub-category and phase of care: absolute values (*N*) and percent distribution (%) within each phase of care

		Initial		Continuing		Final	
		*N*	%	*N*		*N*	%
Category	Sub-category						
Diagnosis and monitoring		90,535	59.8	380,107	92.4	22,464	**73**.4
	Specialist examination	39,042	25.8	147,315	35.8	10,060	32.9
	High diagnostic	12,915	8.5	34,454	8.4	4809	15.7
	Ultrasonography	12,371	8.2	68,218	16.6	1319	4.3
	Conventional radiology	9599	6.3	75,889	18.4	2997	9.8
	Biopsy	6798	4.5	10,261	2.5	344	1.1
	Cardiologic assessment	5736	3.8	28,387	6.9	2126	6.9
	Physiatry	1499	1.0	5929	1.4	631	2.1
	Genetic tests	952	0.6	1427	0.3	115	0.4
	Bone Densitometry	1623	1.1	8227	2.0	63	0.2
Radiotherapy		41,597	27.5	9210	2.2	2711	8.9
Chemotherapy		9407	6.2	9323	2.3	3019	9.9
Post-surgical procedure		3913	2.6	4503	1.1	546	1.8
Hormone therapy		2580	1.7	2306	0.6	555	1.8
Support therapy		1983	1.3	3591	0.9	942	3.1
Psychotherapy		1018	0.7	1685	0.4	103	0.3
Plastic surgery		268	0.2	672	0.2	5	0.0
Transfusion		4	0.0	98	0.0	248	0.8
Total		151,305	100.0	411,495	100.0	30,593	100.0

**Table 3:** Distribution of pharmacy drug prescriptions by category and phase of care: absolute values (*N*) and percent distribution (%) within each phase of care

	Initial		Continuing		Final	
Category	*N*	%	*N*	%	*N*	%
Hormone therapy	18,947	63.5	93,373	67.5	2451	14.1
Cortisone	4246	14.2	15,493	11.2	6684	38.3
Antiemetic	2884	9.7	1579	1.1	844	4.8
Analgesic	1568	5.3	12,4	9.0	6860	39.3
Myelopoietic growth factor	1115	3.7	435	0.3	166	1.0
Bisphosphonate	928	3.1	14,247	10.3	288	1.7
Chemotherapic drug	136	0.5	722	0.5	94	0.5
Hematopoietic growth factors	31	0.1	83	0.1	49	0.3
Total	29,855	100	138	100	17	100

Details of the HD database are illustrated in Table 1, where each hospitalization is classified according to the main cancer-related intervention/procedure. In 12 months, our cohort of patients experienced 24,480 hospitalizations, nearly half of which (11,866) occurred during the initial phase of care, 36% (8900) in continuing phase and 15% (3714) in final phase.

In initial phase, *surgery* is the most frequent procedural category (with conservative surgery—lumpectomy—prevailing over the other surgeries), followed by *chemotherapy* and *diagnosis and monitoring*. In continuing phase *diagnosis and monitoring* category is the most frequent, followed by *surgery* (with plastic surgery prevailing) and *chemotherapy*. In final phase, *diagnosis and monitoring* is the most frequent category, followed by *chemotherapy* and *support therapy*. Within the *diagnosis and monitoring* category, the most frequent sub-category is *biopsy* in initial phase, *cardiologic assessment* in continuing phase and *high diagnostic* in final phase.

Table A2.1 describes the ten most frequent single cancer-related procedures/interventions undertaken during hospitalization, percentages are computed over the total of cancer-related procedures in each phase of care. In initial phase resection of quadrant of breast, chemotherapy and excision of axillary lymph node are the most frequent procedures accounting for about 35.5% of the total; in continuing phase chemotherapy represents the second most frequent procedure followed by diagnostic and monitoring procedures (Electrocardiogram, Routine chest X-rays); a similar pattern holds in final phase.

Details of the OPS database are reported in Table 2. Notice that standard blood tests (35 codes) and genetic markers (9 codes) although included in the D-list of Outpatient services are excluded from the frequency distribution of Table 2: they are individually prescribed (one test corresponds to one record) and account for a huge amount of records, so their inclusion in Table 2 would have biased the frequency distribution.

A total of 593,393 outpatient procedures were administered to our cohort of patients in 12 months: 25% (151,305) in initial phase, 70% (411,495) in continuing phase and 5% (30,593) in final phase. In initial phase, nearly 60% of procedures belong to the *diagnosis and monitoring* category (with *specialist examination* being the most frequent), followed by *radiotherapy* and *chemotherapy*. The same pattern is observed in the remaining phases, with varying percentages: 92.4% of *diagnosis and monitoring* procedures (*specialist examination*, *radiology* and *ultrasonography*) in continuing phase; 73.4% of *diagnosis and monitoring* procedures (*specialist examination*, *high diagnostic*, *radiology* being the more prevalent) in final phase.

Looking at the ten most frequent outpatient procedures by phase of care reported in Table A2.2: check-up examinations is in the first place in all three phases; initial and final phases of care have the same pattern (including chemotherapy, cardiologic assessment, high diagnostic) except for radiotherapy, which is present in initial phase only. In continuing phase, all procedures belong to the category diagnosis and monitoring (i.e., check-up examinations, conventional radiology, cardiologic evaluation, ultrasonography and DXA).

Table 3 describes the distribution of pharmacy drug prescriptions (i.e., drugs prescribed to a patient and sold by a pharmacy).

The most prescribed drugs in initial phase are hormone therapies, followed by cortisone-based drugs and anti-emetics, together representing 87% of drug prescriptions. In continuing phase, hormone therapy is followed by analgesics and cortisone-based drugs (together they represent 88% of total prescriptions). In final phase, analgesics are the most prescribed drugs, followed by cortisone-based drugs and hormone therapy (together representing 92% of prescriptions). Anti-emetics are present mainly in initial and final phases (9.7 and 4.8%, respectively), and are most likely related to chemotherapy. Notice that chemotherapy drugs are poorly represented in this database, which describes only occurrences of drugs sold in a pharmacy. Instead, they can be found in HP database (high cost drugs administered to a patient in hospital), and as procedures in HD (hospital) and OPS (outpatient) databases.

Table A3.3 shows the lists of the ten most frequently drugs sold by pharmacies and reported in the DP database. In initial and continuing phases an anti-estrogen (Tamoxifen) is in the first place, followed by two aromatase inhibitors (Letrozole and Anastrozole). Two bi-phosphonate drugs used for bone diseases (Alendronic Acid and Risedronic Acid) are present in continuing phase only. In final phase, the most frequent drug is a cortisone-based drugs (Dexamethasone), followed by an analgesic (Fentanyl) and another cortisone-based drug (Prednisone).

**Fig. 1:**
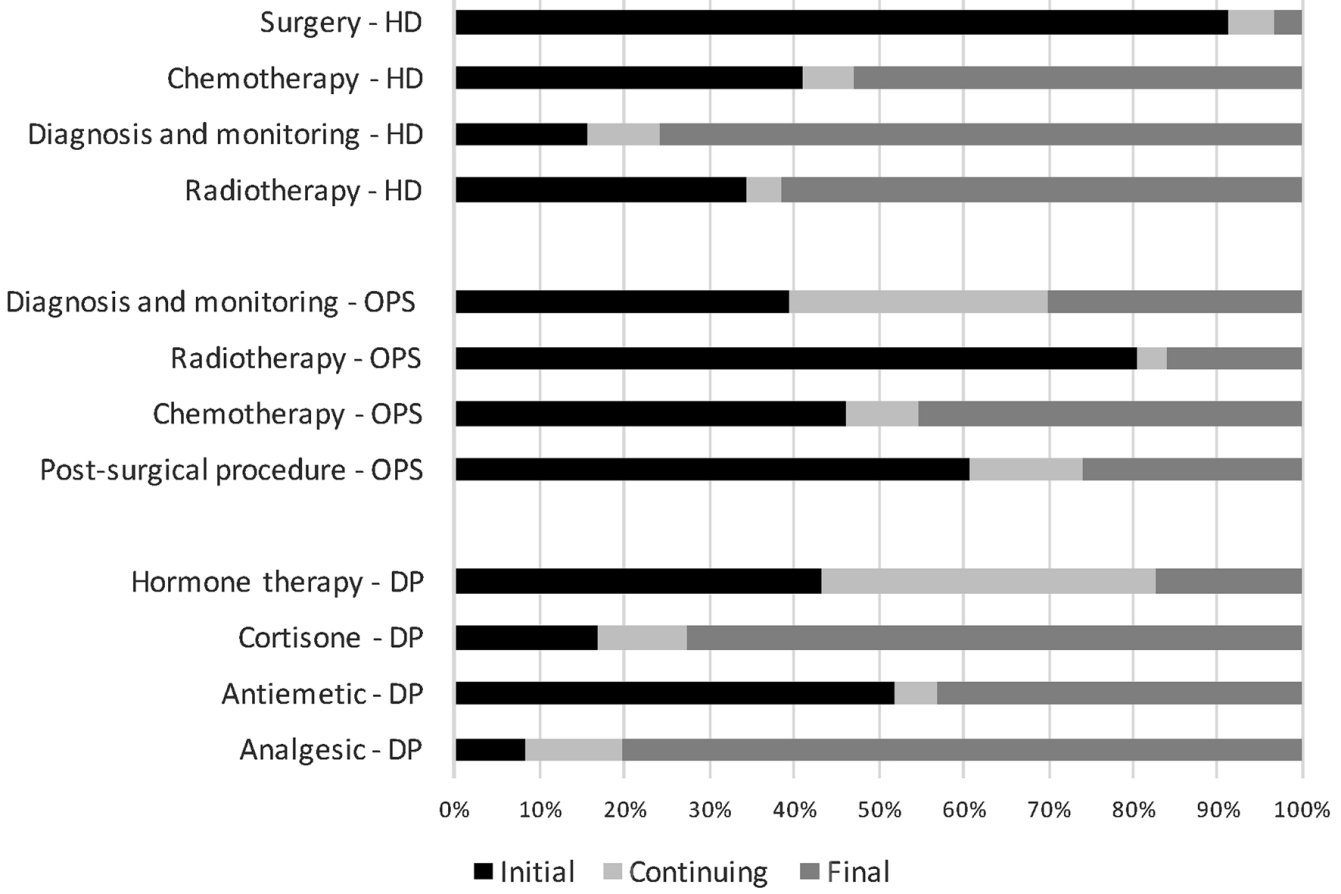
Percent distribution by phases of care of four main categories in each data source: Hospital (HD), outpatient (OPS), pharmacy drugs (DP)

Figure 1 illustrates the distribution by phase of care of the four most frequent (covering at least 90% of total occurrences) hospital and outpatient categories of procedures and pharmacy drug prescriptions; each category is weighted by the percent distribution of patients by phase of care.

Surgery is performed only in hospital setting and mainly during initial phase (91%). Radiotherapy in hospital setting is mostly administered in final phase (62%) while in outpatients setting is mostly administered in initial phase (80%). Chemotherapy in hospital is delivered more frequently in final phase (53%) than in initial phase (41%), while in outpatient setting is equally distributed between initial and final phases. Diagnostic procedures performed in hospital are more frequent in final phase (76%), while in outpatient setting are equally distributed between the three phases. Regarding pharmacy drug prescriptions, distribution patterns vary: hormone therapy is equally distributed between initial and continuing phase; antiemetic drugs are more frequently used in initial and final phase; cortisone-based drugs and analgesics are largely prescribed in final phase.

## Discussion

This paper introduces a direct method consisting of lists of healthcare services related to diagnosis, treatment and follow-up of women with breast cancer. These lists are identified using clinical criteria and used for the estimation of the expenditures directly related to the cancer diagnosis.

This method has already been introduced in cost-of-illness studies, the novelty here is its application to three separated phases of the clinical pathway, from diagnosis to possible recovery or death.

The distribution by phase of care of the occurrences of the D-lists procedures and drug prescriptions is coherent with the patient clinical pathway: surgery is the main cause of hospitalization in the first year since diagnosis; diagnostic and monitoring interventions are more frequent in the following years (to detect possible recurrences), and at the end of life (to arrange palliative care); most ambulatory services belong to diagnosis and monitoring category; one third of services in the initial phase is radiotherapy and chemotherapy; drugs prescribed to patients and sold in pharmacy include hormone therapy in initial and continuing phase and analgesics in final phase.

The D-lists include specific codes related to breast cancer, as well as more generic ones, such as *transfusion* or *support therapy* or drugs classified as *analgesics* or *anti-emetics*. This inclusive approach allows to reduce the risk of under-reporting cancer-related events, with the consequent under-estimation of medical expenditures indirectly associated with breast cancer [3].

The D-lists provided in this paper have been compared to the C-lists obtained by applying the matched control method that uses patients before the diagnosis of breast cancer as self-controls. There is over 97% of concordance; the very few exceptions correspond to: (i) procedures and prescriptions that have not being administered to our cohort of patients, neither before nor after the cancer diagnosis; these codes are nevertheless included in the lists according to clinical criteria; (ii) generic procedures and prescriptions that occur before as well as after the cancer diagnosis (for example X-rays or cortisone); however, since our cohort is made of cancer patients, we assume that after diagnosis they were prescribed because of the cancer; (iii) cancer-related procedures and prescriptions that appear before cancer diagnosis, because for a few patients the period of pre-cancer investigation (which we excluded from the comparison) was longer than 2 months; notice that the choice of the 2-month cut off is appropriate for the large majority of cases. There are some limitations in this work.

The same lists of codes have been used for all phases of care. However, some less specific codes (for example Electrocardiogram or Routine chest X-rays) may be used for more than one chronic condition, and may not be completely suitable for elderly patients with other comorbidities, and in the continuing phase, which includes a mixture of patients with different clinical patterns: patients cured, patients with relapses and patients in chronic condition. A further improvement of the methodology would be to apply differential lists of codes according to clinical variables that possibly influence the patterns of care, such as phase of care, age and stage at diagnosis.

The D-lists are applied to cases diagnosed in the period Jan 1st 2003 to Dec 31st 2010 and refer to treatments administered in that period. Since then there has been a great improvement in drugs, especially immune-therapies based on chimeric monoclonal antibodies (such as Atezolizumab, Pembrolizumab or Pertuzumab), enzyme inhibitors (such as Abemaciclib, Olaparib, or Everolimus), protein-bound chemotherapies (such as Nab-paclitaxel), or other antineoplastic drugs (such as Eribulin). These drugs are not included in the present D-list and their inclusion is recommended in a study based on more recent data.

Finally, the development of the D-lists is quite time consuming and requires the engagement of a panel of experts. Notwithstanding, it should be emphasized that, based on the work produced in this study, the effort required for the revision of the classification system, for the update of the lists or for the adaptation of the lists to other cancer sites, would be minimal.

The approach presented has several strengths.

Firstly, the identification of cancer-related events allows us to describe their occurrences across phases of care, to identify specific cost drivers and, consequently, to suggest best strategies in a public health framework. For example, costs of a chemotherapy session provided in hospital setting is more than five-fold compared to outpatient setting.

Secondly, the direct approach does not require a comparison cohort (which in Italy is not available to CRs), thus avoiding possible biases in the results due to mismatches between cases and controls: indeed, a satisfying degree of matching might be difficult to obtain when dealing with chronic diseases with multiple risk factors that are common to other pathological conditions; moreover, information on risk factors are rarely included in administrative databases such as those used in the present study.

Thirdly, health services in Italy are administered on regional basis and health care databases are available in all regions, in a standardized format. The D-lists based on data from the eight regions included in the Epicost study can be easily applied to other Italian regions, thus producing standardized and highly comparable results. The only adjustment required is the integration of regional-specific codes used for outpatient services.

Finally, the lists of codes developed here are based on international classification systems and can be easily applicable to other countries. As an example, in the ongoing Innovative Partnership for Action Against Cancer (iPAAC) financed by the European Commission the methodology has been proposed for application to other European countries, such as Belgium, Spain, Norway and Poland [15].

## Supplementary Information

Below is the link to the electronic supplementary material.

Supplementary file1 (DOCX 71 kb)

Supplementary file2 (DOCX 30 kb)

Supplementary file3 (DOCX 80 kb)

## Data Availability

Dataset supporting our findings is available, according to AIRTUM guidelines, at the following website: www.registri-tumori.it.
