# Supplementary material for: A direct method for the identification of patterns of care using administrative databases: the case of breast cancer
Source: Eur J Health Econ. 2021 Jul 26;22(9):1477–85. doi: 10.1007/s10198-021-01327-8 (PMC8558165; doi:10.1007/s10198-021-01327-8)
Supplement: Supplementary file 1 — Supplementary file1 (DOCX 71 kb) [file 10198_2021_1327_MOESM1_ESM.docx]

**European Journal of Health Economics**

**Title: A direct method for the identification of patterns of care using administrative databases: the case of breast cancer**

**Appendix 1**. List of breast cancer-related codes

**Tab. A.1.1** - D-list of cancer-related diagnoses and procedures in the Hospital Discharges (HD) database. Codes classified according to the ICD9-CM classification system

**DIAGNOSTIC CODES**

| **ICD9-CM CODE** | **DESCRIPTION** | **CATEGORY** | **SUBCATEGORY** |
| --- | --- | --- | --- |
| 174 | Malignant neoplasm of female breast | Diagnosis and monitoring | Diagnosis |
| 174.0 | Malignant neoplasm of nipple and areola of female breast | Diagnosis and monitoring | Diagnosis |
| 174.1 | Malignant neoplasm of central portion of female breast | Diagnosis and monitoring | Diagnosis |
| 174.2 | Malignant neoplasm of upper-inner quadrant of female breast | Diagnosis and monitoring | Diagnosis |
| 174.3 | Malignant neoplasm of lower-inner quadrant of female breast | Diagnosis and monitoring | Diagnosis |
| 174.4 | Malignant neoplasm of upper-outer quadrant of female breast | Diagnosis and monitoring | Diagnosis |
| 174.5 | Malignant neoplasm of lower-outer quadrant of female breast | Diagnosis and monitoring | Diagnosis |
| 174.6 | Malignant neoplasm of axillary tail of female breast | Diagnosis and monitoring | Diagnosis |
| 174.8 | Malignant neoplasm of other specified sites of female breast | Diagnosis and monitoring | Diagnosis |
| 174.9 | Malignant neoplasm of breast (female), unspecified | Diagnosis and monitoring | Diagnosis |
| 196 | Secondary and unspecified malignant neoplasm of lymph nodes | Diagnosis and monitoring | Diagnosis |
| 196.0 | Secondary and unspecified malignant neoplasm of lymph nodes of head, face, and neck | Diagnosis and monitoring | Diagnosis |
| 196.1 | Secondary and unspecified malignant neoplasm of intrathoracic lymph nodes | Diagnosis and monitoring | Diagnosis |
| 196.2 | Secondary and unspecified malignant neoplasm of intra-abdominal lymph nodes | Diagnosis and monitoring | Diagnosis |
| 196.3 | Secondary and unspecified malignant neoplasm of lymph nodes of axilla and upper limb | Diagnosis and monitoring | Diagnosis |
| 196.5 | Secondary and unspecified malignant neoplasm of lymph nodes of inguinal region and lower limb | Diagnosis and monitoring | Diagnosis |
| 196.6 | Secondary and unspecified malignant neoplasm of intra-pelvic lymph nodes | Diagnosis and monitoring | Diagnosis |
| 196.8 | Secondary and unspecified malignant neoplasm of lymph nodes of multiple sites | Diagnosis and monitoring | Diagnosis |
| 196.9 | Secondary and unspecified malignant neoplasm of lymph nodes, site unspecified | Diagnosis and monitoring | Diagnosis |
| 197 | Secondary malignant neoplasm of respiratory and digestive systems | Diagnosis and monitoring | Diagnosis |
| 197.0 | Secondary malignant neoplasm of lung | Diagnosis and monitoring | Diagnosis |
| 197.1 | Secondary malignant neoplasm of mediastinum | Diagnosis and monitoring | Diagnosis |
| 197.2 | Secondary malignant neoplasm of pleura | Diagnosis and monitoring | Diagnosis |
| 197.3 | Secondary malignant neoplasm of other respiratory organs | Diagnosis and monitoring | Diagnosis |
| 197.4 | Secondary malignant neoplasm of small intestine including duodenum | Diagnosis and monitoring | Diagnosis |
| 197.5 | Secondary malignant neoplasm of large intestine and rectum | Diagnosis and monitoring | Diagnosis |
| 197.6 | Secondary malignant neoplasm of retroperitoneum and peritoneum | Diagnosis and monitoring | Diagnosis |
| 197.7 | Malignant neoplasm of liver, secondary | Diagnosis and monitoring | Diagnosis |
| 197.8 | Secondary malignant neoplasm of other digestive organs and spleen | Diagnosis and monitoring | Diagnosis |
| 198 | Secondary malignant neoplasm of other specified sites | Diagnosis and monitoring | Diagnosis |
| 198.0 | Secondary malignant neoplasm of kidney | Diagnosis and monitoring | Diagnosis |
| 198.1 | Secondary malignant neoplasm of other urinary organs | Diagnosis and monitoring | Diagnosis |
| 198.2 | Secondary malignant neoplasm of skin | Diagnosis and monitoring | Diagnosis |
| 198.3 | Secondary malignant neoplasm of brain and spinal cord | Diagnosis and monitoring | Diagnosis |
| 198.4 | Secondary malignant neoplasm of other parts of nervous system | Diagnosis and monitoring | Diagnosis |
| 198.5 | Secondary malignant neoplasm of bone and bone marrow | Diagnosis and monitoring | Diagnosis |
| 198.6 | Secondary malignant neoplasm of ovary | Diagnosis and monitoring | Diagnosis |
| 198.7 | Secondary malignant neoplasm of adrenal gland | Diagnosis and monitoring | Diagnosis |
| 198.8 | Secondary malignant neoplasm of other specified sites | Diagnosis and monitoring | Diagnosis |
| 198.81 | Secondary malignant neoplasm of breast | Diagnosis and monitoring | Diagnosis |
| 198.82 | Secondary malignant neoplasm of genital organs | Diagnosis and monitoring | Diagnosis |
| 198.89 | Secondary malignant neoplasm of other specified sites | Diagnosis and monitoring | Diagnosis |
| 233.0 | Carcinoma in situ of breast | Diagnosis and monitoring | Diagnosis |
| 238.3 | Neoplasm of uncertain behavior of breast | Diagnosis and monitoring | Diagnosis |
| 284.1 | Pancytopenia | Diagnosis and monitoring | Other diagnostic procedures |
| 288.03 | Drug induced neutropenia | Diagnosis and monitoring | Other diagnostic procedures |
| 338.3 | Neoplasm related pain (acute) (chronic) | Diagnosis and monitoring | Other diagnostic procedures |
| 528.01 | Mucosite post CT | Diagnosis and monitoring | Other diagnostic procedures |
| 733.13 | Pathologic fracture of vertebrae | Diagnosis and monitoring | Other diagnostic procedures |
| 733.15 | Pathologic fracture of other specified part of femur | Diagnosis and monitoring | Other diagnostic procedures |
| 996.54 | Mechanical complication due to breast prosthesis | Diagnosis and monitoring | Other diagnostic procedures |
| V10.3 | Personal history of malignant neoplasm of breast | Diagnosis and monitoring | Diagnosis |
| V43.82 | Breast replacement | Diagnosis and monitoring | Other diagnostic procedures |
| V45.83 | Breast implant removal status | Surgery | Surgery |
| V50.41 | Prophylactic breast removal | Surgery | Surgery |
| V51 | Aftercare involving the use of plastic surgery | Surgery | Plastic surgery |
| V52.4 | Fitting and adjustment of breast prosthesis and implant | Surgery | Plastic surgery |
| V58.0 | Encounter for radiotherapy | Radiotherapy | Radiotherapy |
| V58.1 | Encounter for antineoplastic chemotherapy and immunotherapy | Chemotherapy | Chemotherapy |
| V58.11 | Encounter for antineoplastic chemotherapy | Chemotherapy | Chemotherapy |
| V58.12 | Encounter for antineoplastic immunotherapy | Chemotherapy | Anti-neoplastic immunotherapy |
| V58.81 | Fitting and adjustment of vascular catheter | Chemotherapy | Totally implantable device |
| V66.2 | Convalescence following chemotherapy | Diagnosis and monitoring | Other diagnostic procedures |
| V86.0 | Estrogen receptor positive status [ER+] | Diagnosis and monitoring | Other diagnostic procedures |
| V86.1 | Estrogen receptor negative status [ER-] | Diagnosis and monitoring | Other diagnostic procedures |

**PROCEDURAL CODES**

| **ICD9-CM CODE** | **DESCRIPTION** | **CATEGORY** | **SUBCATEGORY** |
| --- | --- | --- | --- |
| 33.26 | Closed [percutaneous] [needle] biopsy of lung | Diagnosis and monitoring | Biopsy |
| 34.04 | Insertion of intercostal catheter for drainage | Support therapy | Support therapy |
|  |  |  |  |
| 34.91 | Thoracentesis | Diagnosis and monitoring | Invasive procedure |
|  |  |  |  |
| 40.11 | Biopsy of lymphatic structure | Diagnosis and monitoring | Biopsy |
| 40.19 | Other diagnostic procedures on lymphatic structures | Diagnosis and monitoring | Biopsy |
| 40.22 | Excision of internal mammary lymph node | Surgery | Lymphadenectomy |
|  |  |  |  |
| 40.23 | Excision of axillary lymph node | Surgery | Lymphadenectomy |
| 40.29 | Simple excision of other lymphatic structure | Surgery | Lymphadenectomy |
| 40.3 | Regional lymph node excision | Surgery | Lymphadenectomy |
| 40.5 | Radical excision of other lymph nodes | Surgery | Lymphadenectomy |
| 40.50 | Radical excision of lymph nodes, not otherwise specified | Surgery | Lymphadenectomy |
| 40.51 | Radical excision of axillary lymph nodes | Surgery | Lymphadenectomy |
|  |  |  |  |
| 40.59 | Radical excision of other lymph nodes | Surgery | Lymphadenectomy |
| 50.11 | Closed (percutaneous) [needle] biopsy of liver | Diagnosis and monitoring | Biopsy |
| 50.12 | Open biopsy of liver | Diagnosis and monitoring | Biopsy |
| 50.19 | Other diagnostic procedures on liver | Diagnosis and monitoring | Biopsy |
| 50.91 | Percutaneous aspiration of liver | Diagnosis and monitoring | Biopsy |
| 54.24 | Closed [percutaneous] [needle] biopsy of intra-abdominal mass | Diagnosis and monitoring | Biopsy |
| 54.91 | Percutaneous abdominal drainage | Diagnosis and monitoring | Invasive procedure |
|  |  |  |  |
| 68.12 | Hysteroscopy | Surgery | Plastic surgery |
| 68.16 | Closed biopsy of uterus | Diagnosis and monitoring | Biopsy |
| 85.0 | Mastotomy | Diagnosis and monitoring | Biopsy |
| 85.11 | Closed [percutaneous] [needle] biopsy of breast | Diagnosis and monitoring | Biopsy |
| 85.12 | Open biopsy of breast | Diagnosis and monitoring | Biopsy |
| 85.19 | Other diagnostic procedures on breast | Diagnosis and monitoring | Biopsy |
| 85.20 | Excision or destruction of breast tissue, not otherwise specified (NOS) | Surgery | Lumpectomy |
| 85.21 | Local excision of lesion of breast | Surgery | Lumpectomy |
| 85.22 | Resection of quadrant of breast | Surgery | Lumpectomy |
| 85.23 | Subtotal mastectomy | Surgery | Surgery |
|  |  |  |  |
| 85.25 | Excision of nipple | Surgery | Lumpectomy |
| 85.33 | Unilateral subcutaneous mastectomy with synchronous implant | Surgery | Radical and reconstructive surgery |
| 85.34 | Other unilateral subcutaneous mastectomy | Surgery | Surgery |
|  |  |  |  |
| 85.35 | Bilateral subcutaneous mastectomy with synchronous implant | Surgery | Radical and reconstructive surgery |
| 85.36 | Other bilateral subcutaneous mastectomy | Surgery | Surgery |
| 85.41 | Unilateral simple mastectomy | Surgery | Surgery |
| 85.42 | Bilateral simple mastectomy | Surgery | Surgery |
| 85.43 | Unilateral extended simple mastectomy | Surgery | Surgery |
| 85.44 | Bilateral extended simple mastectomy | Surgery | Surgery |
| 85.45 | Subtotal mastectomy | Surgery | Surgery |
| 85.46 | Bilateral radical mastectomy | Surgery | Surgery |
| 85.47 | Unilateral extended radical mastectomy | Surgery | Surgery |
| 85.48 | Bilateral extended radical mastectomy | Surgery | Surgery |
| 85.50 | Augmentation mammoplasty, not otherwise specified | Surgery | Plastic surgery |
| 85.51 | Unilateral injection into breast for augmentation | Surgery | Plastic surgery |
| 85.52 | Bilateral injection into breast for augmentation | Surgery | Plastic surgery |
| 85.53 | Unilateral breast implant | Surgery | Plastic surgery |
| 85.54 | Bilateral breast implant | Surgery | Plastic surgery |
| 85.6 | Mastopexy | Surgery | Plastic surgery |
| 85.7 | Total reconstruction of breast | Surgery | Plastic surgery |
| 85.81 | Suture of breast laceration | Surgery | Plastic surgery |
| 85.82 | Split-thickness graft to breast | Surgery | Plastic surgery |
| 85.83 | Full-thickness graft to breast | Surgery | Plastic surgery |
| 85.84 | Pedicle graft to breast | Surgery | Plastic surgery |
| 85.85 | Muscle flap graft to breast | Surgery | Plastic surgery |
| 85.86 | Transposition of nipple | Surgery | Plastic surgery |
| 85.87 | Other repair or reconstruction of nipple | Surgery | Plastic surgery |
| 85.89 | Other type of mammoplasty | Surgery | Plastic surgery |
| 85.91 | Aspiration of breast | Diagnosis and monitoring | Biopsy |
| 85.93 | Revision of implant of breast | Surgery | Plastic surgery |
| 85.94 | Removal of implant of breast | Surgery | Plastic surgery |
| 85.95 | Insertion of breast tissue expander | Surgery | Plastic surgery |
| 85.96 | Removal of breast tissue expander | Surgery | Plastic surgery |
|  |  |  |  |
| 85.99 | Other operations on the breast | Surgery | Surgery |
|  |  |  |  |
| 86.07 | Insertion of totally implantable vascular access device [VAD] | Chemotherapy | Chemotherapy |
| 86.11 | Biopsy of skin and subcutaneous tissue | Diagnosis and monitoring | Biopsy |
| 86.60 | Free skin graft, not otherwise specified | Surgery | Plastic surgery |
| 87.03 | Computerized axial tomography of head | Diagnosis and monitoring | High diagnostic |
| 87.04 | Other tomography of head | Diagnosis and monitoring | Conventional radiology |
| 87.17 | Other x-ray of skull | Diagnosis and monitoring | Conventional radiology |
| 87.22 | Other x-ray of cervical spine | Diagnosis and monitoring | Conventional radiology |
| 87.23 | Other x-ray of thoracic spine | Diagnosis and monitoring | Conventional radiology |
| 87.24 | Other x-ray of lumbosacral spine | Diagnosis and monitoring | Conventional radiology |
| 87.29 | Other x-ray of spine | Diagnosis and monitoring | Conventional radiology |
| 87.37 | Other mammography | Diagnosis and monitoring | Conventional radiology |
| 87.38 | Sinogram of chest wall | Diagnosis and monitoring | High diagnostic |
| 87.41 | Computerized axial tomography (CAT) of thorax | Diagnosis and monitoring | High diagnostic |
| 87.43 | X-ray of ribs, sternum, and clavicle | Diagnosis and monitoring | Conventional radiology |
| 87.44 | Routine chest x-ray, so described | Diagnosis and monitoring | Conventional radiology |
| 88.01 | Computerized axial tomography (CAT) of abdomen | Diagnosis and monitoring | High diagnostic |
| 88.02 | Other abdomen tomography | Diagnosis and monitoring | Conventional radiology |
| 88.21 | Skeletal x-ray of shoulder and upper arm | Diagnosis and monitoring | Conventional radiology |
| 88.26 | Other skeletal x-ray of pelvis and hip | Diagnosis and monitoring | Conventional radiology |
| 88.27 | Skeletal x-ray of thigh, knee, and lower leg | Diagnosis and monitoring | Conventional radiology |
| 88.31 | Skeletal series | Diagnosis and monitoring | Conventional radiology |
| 88.33 | Other skeletal x-ray | Diagnosis and monitoring | Conventional radiology |
| 88.38 | Other computerized axial tomography (CAT) | Diagnosis and monitoring | High diagnostic |
| 88.72 | Diagnostic ultrasound of heart | Diagnosis and monitoring | Cardiologic assessment |
| 88.73 | Diagnostic ultrasound of other sites of thorax | Diagnosis and monitoring | Ultrasonography |
| 88.74 | Diagnostic ultrasound of digestive system | Diagnosis and monitoring | Ultrasonography |
| 88.75 | Diagnostic ultrasound of urinary system | Diagnosis and monitoring | Ultrasonography |
| 88.76 | Diagnostic ultrasound of abdomen and retroperitoneum | Diagnosis and monitoring | Ultrasonography |
| 88.79 | Other diagnostic ultrasound | Diagnosis and monitoring | Ultrasonography |
| 88.91 | Magnetic resonance imaging of brain and brain stem | Diagnosis and monitoring | High diagnostic |
| 88.92 | Magnetic resonance imaging of chest and myocardium | Diagnosis and monitoring | High diagnostic |
| 88.93 | Magnetic resonance imaging of spinal canal | Diagnosis and monitoring | High diagnostic |
| 88.94 | Magnetic resonance imaging of musculoskeletal | Diagnosis and monitoring | High diagnostic |
| 88.95 | Magnetic resonance imaging of pelvis, prostate, and bladder | Diagnosis and monitoring | High diagnostic |
| 88.97 | Magnetic resonance imaging of other and unspecified sites | Diagnosis and monitoring | High diagnostic |
| 88.98 | Bone mineral density studies | Diagnosis and monitoring | Conventional radiology |
| 89.04 | Other interview and evaluation | Diagnosis and monitoring | Diagnosis |
| 89.52 | Electrocardiogram | Diagnosis and monitoring | Cardiologic assessment |
| 92.14 | Bone scan | Diagnosis and monitoring | High diagnostic |
| 92.16 | Scan of lymphatic system | Diagnosis and monitoring | High diagnostic |
| 92.18 | Total body scan | Diagnosis and monitoring | High diagnostic |
| 92.19 | Total body scan | Diagnosis and monitoring | High diagnostic |
| 92.2 | Therapeutic radiology and nuclear medicine | Radiotherapy | Radiotherapy |
| 92.20 | Infusion of liquid brachytherapy radioisotope | Radiotherapy | Radiotherapy |
| 92.21 | Superficial radiation | Radiotherapy | Radiotherapy |
| 92.22 | Ortho-voltage radiation | Radiotherapy | Radiotherapy |
| 92.23 | Radio-isotopic tele-radiotherapy | Radiotherapy | Radiotherapy |
| 92.24 | Tele-radiotherapy using photons | Radiotherapy | Radiotherapy |
| 92.25 | Tele-radiotherapy using electrons | Radiotherapy | Radiotherapy |
| 92.26 | Tele-radiotherapy of other particulate radiation | Radiotherapy | Radiotherapy |
| 92.27 | Implantation or insertion of radioactive elements | Radiotherapy | Radiotherapy |
| 92.28 | Injection or instillation of radioisotopes | Radiotherapy | Radiotherapy |
| 92.29 | Other radio-therapeutic procedure | Radiotherapy | Radiotherapy |
| 92.3 | Stereotactic radiosurgery | Radiotherapy | Radiotherapy |
| 92.30 | Stereotactic radiosurgery, not otherwise specified | Radiotherapy | Radiotherapy |
| 92.31 | Single source photon radiosurgery | Radiotherapy | Radiotherapy |
| 92.32 | Multi-source photon radiosurgery | Radiotherapy | Radiotherapy |
| 92.33 | Particulate radiosurgery | Radiotherapy | Radiotherapy |
| 92.39 | Stereotactic radiosurgery, not elsewhere classified | Radiotherapy | Radiotherapy |
| 99.0 | Transfusion of blood and blood components | Transfusion | Transfusion |
| 99.00 | Perioperative autologous transfusion of whole blood or blood components | Transfusion | Transfusion |
| 99.02 | Transfusion of previously collected autologous blood | Transfusion | Transfusion |
| 99.03 | Other transfusion of whole blood | Transfusion | Transfusion |
|  | Transfusion: |  |  |
| 99.05 | Transfusion of platelets | Transfusion | Transfusion |
| 99.1 | Injection or infusion of therapeutic or prophylactic substance | Support therapy | Support therapy |
| 99.15 | Parenteral infusion of concentrated nutritional substances | Support therapy | Support therapy |
| 99.22 | Injection of other anti-infective | Support therapy | Support therapy |
| 99.23 | Injection of steroid | Support therapy | Support therapy |
| 99.24 | Injection of other hormone | Support therapy | Support therapy |
| 99.25 | Injection or infusion of cancer chemotherapeutic substance | Chemotherapy | Chemotherapy |
| 99.28 | Injection or infusion of biological response modifier [BRM] as an antineoplastic agent | Biologic therapy | Biologic therapy |
| 99.29 | Injection or infusion of other therapeutic or prophylactic substance | Support therapy | Support therapy |
| 99.85 | Hyperthermia for treatment of cancer | Radiotherapy | Radiotherapy |

**Tab. A.1.2** - D-list of cancer-related procedures in the Outpatient Services (OPS) database. Codes classified according to the ICD9-CM classification system

| **ICD9-CM CODE** | **DESCRIPTION** | **CATEGORY** | **SUBCATEGORY** |
| --- | --- | --- | --- |
| 03.8 | Injection of cytotoxic drug into the vertebral canal | Chemotherapy | Chemotherapy |
| 33.26 | [Percutaneous] [needle] biopsy of lung | Diagnosis and monitoring | Biopsy |
| 34.91 | Thoracentesis | Support therapy | Support therapy |
| 34.91.1 | Thoracentesis; CAT-guided | Support therapy | Support therapy |
| 38.93.2 | Central venous catheterization. Includes: follow-up x-rays | Post-surgical procedure | Post-surgical procedure |
| 38.99.1 | Contrast material administration for MRI radiotherapy simulation | Radiotherapy | Radiotherapy |
| 38.99.2 | Contrast material administration for CT radiotherapy simulation | Radiotherapy | Radiotherapy |
| 40.11 | Open biopsy of lymphatic structures: cervical, supraclavicular or supraclavicular lymph nodes; axillary lymph nodes | Diagnosis and monitoring | Biopsy |
| 40.19.1 | Ultrasound-guided fine needle biopsy of lymph node | Diagnosis and monitoring | Biopsy |
| 40.19.2 | CT-guided fine needle biopsy of lymph node | Diagnosis and monitoring | Biopsy |
| 50.11 | Closed (percutaneous) [needle] biopsy of liver | Diagnosis and monitoring | Biopsy |
| 50.19.1 | [Percutaneous] [needle] biopsy of liver | Diagnosis and monitoring | Biopsy |
| 50.91 | Percutaneous aspiration of liver | Diagnosis and monitoring | Biopsy |
| 50.91.1 | Ultrasound guided percutaneous aspiration of liver cyst | Diagnosis and monitoring | Biopsy |
| 54.24 | Closed [percutaneous] [needle] biopsy of intra-abdominal mass | Diagnosis and monitoring | Biopsy |
| 54.24.1 | [Percutaneous] [needle] Ultrasound guided biopsy of intra-abdominal mass | Diagnosis and monitoring | Biopsy |
| 54.91 | Percutaneous abdominal drainage | Post-surgical procedure | Post-surgical procedure |
| 54.91.1 | CAT-guided percutaneous abdominal drainage | Post-surgical procedure | Post-surgical procedure |
| 68.12.1 | Hysteroscopy | Plastic surgery | Plastic surgery |
| 68.16.1 | Closed biopsy of uterus | Diagnosis and monitoring | Biopsy |
| 83.21 | Open biopsy of soft tissue | Diagnosis and monitoring | Biopsy |
| 83.21.1 | Ultrasound-guided biopsy of soft tissue | Diagnosis and monitoring | Biopsy |
| 85.0 | MASTOTOMY; Incision of breast (skin) | Diagnosis and monitoring | Biopsy |
| 85.11 | Closed [percutaneous] [needle] biopsy of breast | Diagnosis and monitoring | Biopsy |
| 85.11.1 | Ultrasound-guided biopsy of breast; | Diagnosis and monitoring | Biopsy |
| 85.2 | Excision or Destruction of Breast Tissue | Diagnosis and monitoring | Biopsy |
| 85.20 | Excision or destruction of breast tissue,NOS. | Diagnosis and monitoring | Biopsy |
| 85.21 | Local excision of lesion of breast; Lumpectomy | Diagnosis and monitoring | Biopsy |
| 85.21.1 | Percutaneous aspiration of breast cists | Diagnosis and monitoring | Biopsy |
| 86.01 | Aspiration of skin and subcutaneous tissue; | Diagnosis and monitoring | Biopsy |
| 86.01.1 | Injection for skin expander adaptation | Plastic surgery | Plastic surgery |
| 86.07 | Insertion of totally implantable vascular access device [VAD] | Chemotherapy | Chemotherapy |
| 86.11 | Closed biopsy of skin and subcutaneous tissue | Diagnosis and monitoring | Biopsy |
| 86.4 | Radical excision of skin lesion | Diagnosis and monitoring | Biopsy |
| 86.60 | Free skin graft, NOS | Plastic surgery | Plastic surgery |
| 87.03 | Computerized axial tomography (CAT) of head | Diagnosis and monitoring | High diagnostics |
| 87.03.1 | Computerized axial tomography (CAT) of head, withouth and with contrast | Diagnosis and monitoring | High diagnostics |
| 87.03.7 | Computerized axial tomography (CAT) of neck | Diagnosis and monitoring | High diagnostics |
| 87.03.8 | Computed axial tomography (CAT) of neck, without and with contrast | Diagnosis and monitoring | High diagnostics |
| 87.17.1 | X-ray of skull and paranasal sinuses | Diagnosis and monitoring | Conventional radiology |
| 87.17.4 | X-ray of skull | Diagnosis and monitoring | Conventional radiology |
| 87.22 | Other x-ray of cervical spine | Diagnosis and monitoring | Conventional radiology |
| 87.23 | Other x-ray of thoracic spine | Diagnosis and monitoring | Conventional radiology |
| 87.24 | Other x-ray of lumbosacral spine | Diagnosis and monitoring | Conventional radiology |
| 87.24.1 | Other x-ray of lumbosacral spine | Diagnosis and monitoring | Conventional radiology |
| 87.24.2 | X-ray of Sacrococcygeal spine | Diagnosis and monitoring | Conventional radiology |
| 87.29 | Other x-ray of spine | Diagnosis and monitoring | Conventional radiology |
| 87.35 | Contrast radiogram of mammary ducts | Diagnosis and monitoring | Conventional radiology |
| 87.37.1 | Bilateral mammography (2 projections) | Diagnosis and monitoring | Conventional radiology |
| 87.37.2 | Mono-lateral mammography (2 projections) | Diagnosis and monitoring | Conventional radiology |
| 87.41 | Computerized axial tomography (CAT) of thorax | Diagnosis and monitoring | High diagnostics |
| 87.41.1 | Computerized axial tomography (CAT) of thorax, without and with contrast | Diagnosis and monitoring | High diagnostics |
| 87.42.1 | Bilateral tomography [stratigraphy] of thorax | Diagnosis and monitoring | High diagnostics |
| 87.42.2 | Unilateral tomography [stratigraphy] of thorax | Diagnosis and monitoring | Conventional radiology |
| 87.42.3 | Tomography [stratigraphy] of mediastinum | Diagnosis and monitoring | Conventional radiology |
| 87.43.1 | Bilateral x-ray of ribs and clavicle (3 projections) | Diagnosis and monitoring | Conventional radiology |
| 87.43.2 | X-ray of ribs, sternum, and clavicle (2 projections); | Diagnosis and monitoring | Conventional radiology |
| 87.44.1 | Routine x-ray of thorax, NOS | Diagnosis and monitoring | Conventional radiology |
| 88.01.1 | Computerized axial tomography (CAT) of upper abdomen. | Diagnosis and monitoring | High diagnostics |
| 88.01.2 | Computerized axial tomography (CAT) of upper abdomen with and without contrast; | Diagnosis and monitoring | High diagnostics |
| 88.01.3 | Computerized axial tomography (CAT) of lower abdomen. | Diagnosis and monitoring | High diagnostics |
| 88.01.4 | Computed tomography (CAT) of lower abdomen, without and with contrast. | Diagnosis and monitoring | High diagnostics |
| 88.01.5 | Computed tomography (CAT) of whole abdomen | Diagnosis and monitoring | High diagnostics |
| 88.01.6 | Computed tomography (CAT) of whole abdomen, with and without contrast | Diagnosis and monitoring | High diagnostics |
| 88.21 | X-ray of shoulder and upper arm | Diagnosis and monitoring | Conventional radiology |
| 88.26 | X-ray of pelvis and hip | Diagnosis and monitoring | Conventional radiology |
| 88.27 | X-ray of thigh, knee, and lower leg (2 projections) | Diagnosis and monitoring | Conventional radiology |
| 88.27.3 | X-ray of lower leg | Diagnosis and monitoring | Conventional radiology |
| 88.31 | X-ray of whole skeleton | Diagnosis and monitoring | Conventional radiology |
| 88.33.2 | Tomography [stratigraphy] of skeletal segment | Diagnosis and monitoring | Conventional radiology |
| 88.38.1 | Computerised axial tomography (CAT) of rachid and vertebral speco; | Diagnosis and monitoring | High diagnostics |
| 88.38.2 | Computerised axial tomography (CAT) of rachid and vertebral speco with and without contrast; | Diagnosis and monitoring | High diagnostics |
| 88.38.3 | Computerised axial tomography (CAT) of shoulder, elbow, wrist and hand; | Diagnosis and monitoring | High diagnostics |
| 88.38.4 | Computerised axial tomography (CAT) of shoulder, elbow, wrist and hand with and without contrast; | Diagnosis and monitoring | High diagnostics |
| 88.38.5 | Computerised axial tomography (CAT) OF pelvis | Diagnosis and monitoring | High diagnostics |
| 88.38.6 | Computerised axial tomography (CAT) of femur, knee, ankle and foot | Diagnosis and monitoring | High diagnostics |
| 88.38.7 | Computerised axial tomography (CAT) of femur, knee, ankle and foot with and without contrast | Diagnosis and monitoring | High diagnostics |
| 88.72.1 | Cardiac ultrasound | Diagnosis and monitoring | Cardiologic assessment |
| 88.73.1 | Bilateral ultrasound of breast | Diagnosis and monitoring | Ultrasonography |
| 88.73.2 | Mono-lateral ultrasound of breast | Diagnosis and monitoring | Ultrasonography |
| 88.73.4 | Breast Doppler echocardiography | Diagnosis and monitoring | Ultrasonography |
| 88.73.6 | Breast biopsy with stereotaxic | Diagnosis and monitoring | Biopsy |
| 88.73.7 | Breast micro-biopsy with stereotaxic technique | Diagnosis and monitoring | Biopsy |
| 88.74.1 | Ultrasound of upper abdomen | Diagnosis and monitoring | Ultrasonography |
| 88.75.1 | Ultrasound of lower abdomen | Diagnosis and monitoring | Ultrasonography |
| 88.76.1 | Ultrasound of whole abdomen | Diagnosis and monitoring | Ultrasonography |
| 88.78.2 | Gynaecological ultrasound | Diagnosis and monitoring | Ultrasonography |
| 88.79.7 | Transvaginal ultrasound | Diagnosis and monitoring | Ultrasonography |
| 88.90.2 | Three-dimensional computerized Axial tomography (CAT) reconstruction | Diagnosis and monitoring | High diagnostics |
| 88.90.3 | Computerized Axial tomography (CAT) of the rachis and the spinal canal | Diagnosis and monitoring | High diagnostics |
| 88.91.1 | Magnetic resonance imaging (MRI) of brain and brain stem | Diagnosis and monitoring | High diagnostics |
| 88.91.2 | Magnetic resonance imaging (MRI) of brain and brain stem, without and with contrast | Diagnosis and monitoring | High diagnostics |
| 88.92 | Magnetic resonance imaging of thorax | Diagnosis and monitoring | High diagnostics |
| 88.92.1 | Magnetic resonance imaging (MRI) of Thorax, without and with contrast | Diagnosis and monitoring | High diagnostics |
| 88.92.6 | Magnetic resonance imaging (MRI) of breast; mono-lateral | Diagnosis and monitoring | High diagnostics |
| 88.92.7 | Magnetic resonance imaging (MRI) of breast; mono-lateral without and with contrast | Diagnosis and monitoring | High diagnostics |
| 88.92.8 | Magnetic resonance imaging (MRI) of breast; bilateral | Diagnosis and monitoring | High diagnostics |
| 88.92.9 | Magnetic resonance imaging (MRI) of breast; bilateral, without and with contrast | Diagnosis and monitoring | High diagnostics |
| 88.93 | Magnetic resonance imaging (MRI) of vertebral column, without and with contrast | Diagnosis and monitoring | High diagnostics |
| 88.93.1 | Magnetic resonance imaging (MRI) of vertebral column, without and with contrast | Diagnosis and monitoring | High diagnostics |
| 88.94.1 | Musculoskeletal magnetic resonance imaging (MRI) | Diagnosis and monitoring | High diagnostics |
| 88.94.2 | Musculoskeletal magnetic resonance imaging (MRI) without and with contrast | Diagnosis and monitoring | High diagnostics |
| 88.95.1 | Magnetic resonance imaging (MRI) of upper abdomen | Diagnosis and monitoring | High diagnostics |
| 88.95.2 | Magnetic resonance imaging (MRI) of upper abdomen, without and with contrast | Diagnosis and monitoring | High diagnostics |
| 88.95.3 | Magnetic resonance angiography (MRA) of upper abdomen | Diagnosis and monitoring | High diagnostics |
| 88.95.4 | Magnetic resonance imaging (MRI) of lower abdomen and pelvic cavity | Diagnosis and monitoring | High diagnostics |
| 88.95.5 | Magnetic resonance imaging (MRI) of lower abdomen and pelvic cavity, without and with contrast | Diagnosis and monitoring | High diagnostics |
| 88.95.6 | Magnetic resonance angiography (MRA) of lower abdomen | Diagnosis and monitoring | High diagnostics |
| 88.99.1 | Bone mineral density studies via single or dual photon absorptiometry of wrist or ankle | Diagnosis and monitoring | Bone Densitometry (BMD) |
| 88.99.2 | Bone mineral density studies via Dual-energy X-ray absorptiometry (DXA) | Diagnosis and monitoring | Bone Densitometry (BMD) |
| 88.99.3 | Bone mineral density studies via Dual-energy X-ray absorptiometry (DXA); total body | Diagnosis and monitoring | Bone Densitometry (BMD) |
| 88.99.4 | Bone mineral density studies, via computed axial tomography (CAT); lumbar | Diagnosis and monitoring | Bone Densitometry (BMD) |
| 88.99.5 | Bone mineral density studies via ultrasound | Diagnosis and monitoring | Bone Densitometry (BMD) |
| 89.01 | Check-up examination, only the following specialties: 09=General surgery; 12=plastic surgery; 61=high diagnostics- nuclear medicine; 64=oncology; 69=radiology; 70=radiotherapy | Diagnosis and monitoring | Specialist examination |
| 89.03 | Check-up examination, described as comprehensive only the following specialties: 09=General surgery; 12=plastic surgery; 61=high diagnostics- nuclear medicine; 64=oncology; 69=radiology; 70=radiotherapy | Diagnosis and monitoring | Specialist examination |
| 89.07 | Consultation, described as comprehensive only the following specialties: 09=General surgery; 12=plastic surgery; 61=high diagnostics- nuclear medicine; 64=oncology; 69=radiology; 70=radiotherapy | Diagnosis and monitoring | Specialist examination |
| 89.52 | Electrocardiogram | Diagnosis and monitoring | Cardiologic assessment |
| 89.7 | First visit only the following specialties: 09=General surgery; 12=plastic surgery; 61=high diagnostics- nuclear medicine; 64=oncology; 69=radiology; 70=radiotherapy | Diagnosis and monitoring | Specialist examination |
| 90.04.5 | Alanine aminotransferase | Diagnosis and monitoring | Blood test |
| 90.05.1 | Albumin | Diagnosis and monitoring | Blood test |
| 90.05.5 | Alpha 1 fetoprotein [S/La/Alb] | Diagnosis and monitoring | Bio-marker |
| 90.07.5 | Ammonium [P] | Diagnosis and monitoring | Blood test |
| 90.09.2 | Aspartate aminotransferase (AST) (GOT) [S] | Diagnosis and monitoring | Blood test |
| 90.10.4 | Total bilirubin | Diagnosis and monitoring | Blood test |
| 90.10.5 | Total and fractionated bilirubin | Diagnosis and monitoring | Blood test |
| 90.11.4 | Total calcium | Diagnosis and monitoring | Blood test |
| 90.13.3 | Chloride | Diagnosis and monitoring | Blood test |
| 90.15.4 | Creatine Kinase | Diagnosis and monitoring | Blood test |
| 90.16.3 | Creatinine [S/U/du/La] | Diagnosis and monitoring | Blood test |
| 90.16.4 | Creatinine CLEARANCE | Diagnosis and monitoring | Blood test |
| 90.19.2 | Estradiol (E2) [S/U] | Diagnosis and monitoring | Blood test |
| 90.23.3 | Follitropin (FSH) [S/U] | Diagnosis and monitoring | Blood test |
| 90.23.5 | Alkaline phosphatase | Diagnosis and monitoring | Blood test |
| 90.24.1 | Alkaline phosphatase | Diagnosis and monitoring | Blood test |
| 90.25.5 | Gamma glutamil transpeptidase (gamma GT) [S/U] | Diagnosis and monitoring | Blood test |
| 90.27.1 | Glucosium [S/P/U/du/La] | Diagnosis and monitoring | Blood test |
| 90.29.2 | Lactate dehydrogenase | Diagnosis and monitoring | Blood test |
| 90.30.4 | Liquid effusions: physical and chemical test | Diagnosis and monitoring | Other test |
| 90.32.3 | Luteotropin (LH) [S/U] | Diagnosis and monitoring | Blood test |
| 90.37.4 | Potassium [S/U/du/(Sg)Er] | Diagnosis and monitoring | Blood test |
| 90.38.1 | Progesterone [S] | Diagnosis and monitoring | Blood test |
| 90.38.4 | Protein electrophoresis | Diagnosis and monitoring | Blood test |
| 90.38.5 | Proteins | Diagnosis and monitoring | Blood test |
| 90.39.5 | Oestrogen receptor | Diagnosis and monitoring | Biopsy |
| 90.40.1 | Progesterone receptor | Diagnosis and monitoring | Biopsy |
| 90.40.4 | Sodium | Diagnosis and monitoring | Blood test |
| 90.44.1 | Urea [S/P/U/du] | Diagnosis and monitoring | Blood test |
| 90.55.1 | CA 125 | Diagnosis and monitoring | Genetic marker |
| 90.55.2 | CA 15.3 | Diagnosis and monitoring | Genetic marker |
| 90.55.3 | CA 19.9 | Diagnosis and monitoring | Genetic marker |
| 90.55.4 | CA 195 | Diagnosis and monitoring | Genetic marker |
| 90.55.5 | CA 50 | Diagnosis and monitoring | Genetic marker |
| 90.56.1 | CA 72-4 | Diagnosis and monitoring | Genetic marker |
| 90.56.2 | MCA | Diagnosis and monitoring | Genetic marker |
| 90.56.3 | Carcinoembryonic antigen | Diagnosis and monitoring | Genetic marker |
| 90.56.4 | TPA | Diagnosis and monitoring | Genetic marker |
| 90.62.2 | Blood count (CBC): Hb, GR, GB, HCT, PLT, IND. DERIV., F. L. | Diagnosis and monitoring | Blood test |
| 90.70.4 | Leukocyte (complete and differential blood count) | Diagnosis and monitoring | Blood test |
| 90.70.5 | Leukocyte | Diagnosis and monitoring | Blood test |
| 90.71.3 | Platelet count | Diagnosis and monitoring | Blood test |
| 90.71.31 | Platelet count | Diagnosis and monitoring | Blood test |
| 90.71.32 | Platelet count | Diagnosis and monitoring | Blood test |
| 90.75.4 | Prothrombin time | Diagnosis and monitoring | Blood test |
| 90.76.1 | Partial Prothrombin time | Diagnosis and monitoring | Blood test |
| 90.94.1 | Blood culture | Diagnosis and monitoring | Blood test |
| 91.29.1 | DNA anlysis and hybridization with molecular tool | Diagnosis and monitoring | Genetic test |
| 91.29.2 | DNA test for polymorphism | Diagnosis and monitoring | Genetic test |
| 91.29.3 | DNA mutation analysis | Diagnosis and monitoring | Genetic test |
| 91.29.4 | DNA mutation analysis | Diagnosis and monitoring | Genetic test |
| 91.29.5 | DNA mutation analysis | Diagnosis and monitoring | Genetic test |
| 91.30.1 | DNA mutation analysis | Diagnosis and monitoring | Genetic test |
| 91.36.1 | Storage of DNA or RNA sample | Diagnosis and monitoring | Genetic test |
| 91.36.3 | Cryo-conservation in liquid nitrogen of cell and tissue | Diagnosis and monitoring | Genetic test |
| 91.36.5 | Extraction of DNA or RNA | Diagnosis and monitoring | Genetic test |
| 91.37.1 | Hybridization with molecular tool | Diagnosis and monitoring | Genetic test |
| 91.37.2 | Hybridization in situ (FISH) | Diagnosis and monitoring | Genetic test |
| 91.37.3 | Hybridization in situ (FISH) | Diagnosis and monitoring | Genetic test |
| 91.37.4 | Hybridization in situ (FISH) | Diagnosis and monitoring | Genetic test |
| 91.37.5 | Hybridization in situ (FISH) | Diagnosis and monitoring | Genetic test |
| 91.38.4 | Analysis of cellular DNA for cytometric study and ploidy analysis | Diagnosis and monitoring | Genetic test |
| 91.39.1 | Fine needle aspiration cytology NOS | Diagnosis and monitoring | Biopsy |
| 91.39.3 | Cytological examination of effusions | Diagnosis and monitoring | Biopsy |
| 91.39.6 | Immuno-histochemical predictive marker of response to therapy; HERCEP-TEST (EGFR) | Diagnosis and monitoring | Biopsy |
| 91.40.4 | Cytopathology biopsy (Skin) | Diagnosis and monitoring | Biopsy |
| 91.40.5 | Excisional biopsy (Skin and/or soft tissue) | Diagnosis and monitoring | Biopsy |
| 91.41.1 | Incisional biopsy (Skin and/or soft tissue) | Diagnosis and monitoring | Biopsy |
| 91.41.2 | Fine needle biopsy of liver | Diagnosis and monitoring | Biopsy |
| 91.44.3 | Cytopathology biopsy of urogenital apparatus (with channel scraping) | Diagnosis and monitoring | Biopsy |
| 91.44.5 | Cytopathology biopsy of urogenital apparatus (Endometrial biopsy) | Diagnosis and monitoring | Biopsy |
| 91.46.5 | Cytopathology biopsy of breast Stereotactic biopsy | Diagnosis and monitoring | Biopsy |
| 91.47.1 | Cytopathology biopsy of breast: Nodulectomy | Diagnosis and monitoring | Biopsy |
| 91.47.2 | Fine needle biopsy of EMOPOIETIC SYSTEM: Lymph node ago-biopsy | Diagnosis and monitoring | Biopsy |
| 91.47.3 | Fine needle biopsy of EMOPOIETIC SYSTEM: Lymph node ago-biopsy (multiple sites) | Diagnosis and monitoring | Biopsy |
| 91.47.4 | Cytopathology biopsy of emopoietic system: Removal of surface lymph node | Diagnosis and monitoring | Biopsy |
| 91.48.4 | Cytological sampling | Diagnosis and monitoring | Biopsy |
| 91.49.2 | Venous blood sampling | Diagnosis and monitoring | Blood test |
| 92.11.5 | Cerebral tomo-scintigraphy | Diagnosis and monitoring | High diagnostics |
| 92.11.6 | Cerebral positron emission tomography (PET) | Diagnosis and monitoring | High diagnostics |
| 92.14.1 | Segmental bone or articular scintigraphy | Diagnosis and monitoring | High diagnostics |
| 92.14.2 | Segmental bone or articular scintigraphy | Diagnosis and monitoring | High diagnostics |
| 92.16.1 | Segmental scintigraphy of lymphatic system | Diagnosis and monitoring | High diagnostics |
| 92.18.1 | Whole (total) body scintigraphy using radioiodine | Diagnosis and monitoring | High diagnostics |
| 92.18.2 | Bone or articular scintigraphy | Diagnosis and monitoring | High diagnostics |
| 92.18.4 | Total body scintigraphy via radio-labelled autologous cells | Diagnosis and monitoring | High diagnostics |
| 92.18.5 | Total body scintigraphy via immunologic and recombinant tracers | Diagnosis and monitoring | High diagnostics |
| 92.18.6 | Total body positron emission tomography (PET) | Diagnosis and monitoring | High diagnostics |
| 92.19.6 | Segmental scintigraphy after total body scintigraphy | Diagnosis and monitoring | High diagnostics |
| 92.19.8 | Total body positron emission tomography (PET) with computerized Axial tomography (CAT) | Diagnosis and monitoring | High diagnostics |
| 92.23.1 | Cobalt therapy with fixed beam or two opposite beams | Radiotherapy | Radiotherapy |
| 92.23.2 | Cobalt therapy with multiple beams | Radiotherapy | Radiotherapy |
| 92.23.3 | Cobalt therapy with flash technique | Radiotherapy | Radiotherapy |
| 92.24.1 | Radiation Therapy using LINAC with direct beam | Radiotherapy | Radiotherapy |
| 92.24.2 | Radiation Therapy using LINAC with multiple beam | Radiotherapy | Radiotherapy |
| 92.24.3 | Radiation Therapy using LINAC with flash technique | Radiotherapy | Radiotherapy |
| 92.24.4 | Stereotactic radiotherapy | Radiotherapy | Radiotherapy |
| 92.25.1 | Electron beam therapy with one or multiple beams | Radiotherapy | Radiotherapy |
| 92.28.4 | Monoclonal antibody therapy; up to 185 mbq | Support therapy | Support therapy |
| 92.28.5 | Monoclonal antibody therapy; for each successive 185 mbq | Support therapy | Support therapy |
| 92.28.6 | Palliative therapy of pain from bone metastasis | Support therapy | Support therapy |
| 92.29.1 | Target acquisition via x-ray simulator | Radiotherapy | Radiotherapy |
| 92.29.2 | Target acquisition via computerised tomography simulator or computerised tomography (CT) | Radiotherapy | Radiotherapy |
| 92.29.3 | Target acquisition via magnetic resonance (MR) | Radiotherapy | Radiotherapy |
| 92.29.4 | Physical/dosimetric evaluation; dose calculation | Radiotherapy | Radiotherapy |
| 92.29.5 | Physical/dosimetric evaluation with computed tomography (CT) scans | Radiotherapy | Radiotherapy |
| 92.29.6 | In vivo dosimetry | Radiotherapy | Radiotherapy |
| 92.29.7 | Customized shielding | Radiotherapy | Radiotherapy |
| 92.29.8 | Personalized immobilization system | Radiotherapy | Radiotherapy |
| 93.01.1 | Global functional evaluation (with psycho-behavioral scale) | Diagnosis and monitoring | Physiatry |
| 93.01.2 | Segmental functional evaluation (with psycho-behavioral scale) | Diagnosis and monitoring | Physiatry |
| 93.04.1 | Manual testing of muscle function; general test on joints and muscles | Diagnosis and monitoring | Physiatry |
| 93.04.2 | Manual testing of muscle function; segmental test on joints and muscles | Diagnosis and monitoring | Physiatry |
| 93.11.1 | Individual (motor)rehabilitation for seriously motion-impaired patient; complex instrumental rehabilitation | Diagnosis and monitoring | Physiatry |
| 93.11.2 | Individual (motor)rehabilitation for seriously motion-impaired patient; simple instrumental rehabilitation | Diagnosis and monitoring | Physiatry |
| 93.11.3 | Individual (motor)rehabilitation for segmental motion-impaired patient; complex instrumental rehabilitation | Diagnosis and monitoring | Physiatry |
| 93.11.4 | Individual (motor)rehabilitation for segmental motion-impaired patient; simple instrumental rehabilitation | Diagnosis and monitoring | Physiatry |
| 93.11.5 | Group motor rehabilitation | Diagnosis and monitoring | Physiatry |
| 93.12.2 | Functional rehabilitation | Diagnosis and monitoring | Physiatry |
| 93.39.2 | Massotherapy for lymphatic drainage | Diagnosis and monitoring | Physiatry |
| 93.40.2 | Massotherapy for lymphatic drainage | Diagnosis and monitoring | Physiatry |
| 94.09 | Psychologic mental status determination | Psychotherapy | Psychotherapy |
| 94.3 | Individual psychotherapy | Psychotherapy | Psychotherapy |
| 94.32 | Hypnotherapy | Psychotherapy | Psychotherapy |
| 94.42 | Other psychotherapy and counselling - Family therapy | Psychotherapy | Psychotherapy |
| 94.44 | Other psychotherapy and counselling - Group therapy | Psychotherapy | Psychotherapy |
| 96.59 | Other irrigation of wound | Post-surgical procedure | Post-surgical procedure |
| 99.07.1 | Transfusion of blood or blood components | Transfusion | Transfusion |
| 99.23 | Injection of steroid; Injection of cortisone; Subdermal implantation of progesterone or other hormone | Hormone therapy | Hormone therapy |
| 99.24.1 | Injection of hormone | Hormone therapy | Hormone therapy |
| 99.25 | Injection or infusion of cancer chemotherapeutic substance; | Chemotherapy | Chemotherapy |
| 99.85 | Hyperthermia for cancer treatment | Radiotherapy | Radiotherapy |

**Tab. A.1.3** - D-list of cancer-related drugs in “Drug prescribed to a patient and sold by a pharmacy” database (DP) and in “Hospital pharmacy” database (HP). Codes classified according to the Anatomic Therapeutic Chemical (ATC) classification system.

| **ATC CODE** | **ACTIVE INGREDIENT** | **CATEGORY** |
| --- | --- | --- |
| A03FA | Levosulpiride | Antiemetic |
| A03FA01 | Metoclopramide | Antiemetic |
| A03FA05 | Alizapride | Antiemetic |
| A04AA01 | Ondansetron | Antiemetic |
| A04AA02 | Granisetrone | Antiemetic |
| A04AA03 | Tropisetron | Antiemetic |
| A04AA05 | Palonosetron hydrochloride | Antiemetic |
| A04AD12 | Aprepitant | Antiemetic |
| A07DA03 | Loperamide | Antiemetic |
| B03XA01 | Epoetin alpha | Hematopoietic growth factor |
| B03XA02 | Darbepoetin alfa | Hematopoietic growth factor |
| H02AB01 | Betamethasone | Cortisone |
| H02AB02 | Dexamethasone | Cortisone |
| H02AB04 | Methylprednisolone | Cortisone |
| H02AB07 | Prednisone | Cortisone |
| H02AB09 | Hydrocortisone | Cortisone |
| H02AB10 | Cortisone | Cortisone |
| L01AA01 | Cyclophosphamide | Chemotherapic drug |
| L01BA01 | Methotrexate | Chemotherapic drug |
| L01BC02 | Fluorouracil | Chemotherapic drug |
| L01BC05 | Gemcitabine | Chemotherapic drug |
| L01BC06 | Capecitabine | Chemotherapic drug |
| L01CA04 | Vinorelbine | Chemotherapic drug |
| L01CD01 | Paclitaxel | Chemotherapic drug |
| L01CD02 | Docetaxel | Chemotherapic drug |
| L01DB01 | Doxorubicin | Chemotherapic drug |
| L01DB02 | Daunorubicin | Chemotherapic drug |
| L01DB03 | Epirubicin | Chemotherapic drug |
| L01XC03 | Trastuzumab | Biologic therapy |
| L01XC07 | Bevacizumab | Biologic therapy |
| L01XC14 | Trastuzumab emtansine | Biologic therapy |
| L01XE07 | Lapatinib | Biologic therapy |
| L02AB01 | Megestrol | Hormone therapy |
| L02AE01 | Buserelin | Hormone therapy |
| L02AE02 | Leuprorelin | Hormone therapy |
| L02AE03 | Goserelin | Hormone therapy |
| L02AE04 | Triptorelin | Hormone therapy |
| L02BA01 | Tamoxifen | Hormone therapy |
| L02BA02 | Toremifene | Hormone therapy |
| L02BA03 | Fulvestrant | Hormone therapy |
| L02BG03 | Anastrozole | Hormone therapy |
| L02BG04 | Letrozole | Hormone therapy |
| L02BG06 | Exemestane | Hormone therapy |
| L03AA02 | Filgrastim | Myelopoietic growth factor |
| L03AA10 | Lenograstim | Myelopoietic growth factor |
| L03AA13 | Pegfilgrastim | Myelopoietic growth factor |
| M05BA | Sodium nerhydronate | Bisphosphonate |
| M05BA04 | Alendronic acid | Bisphosphonate |
| M05BA06 | Ibandronic acid | Bisphosphonate |
| M05BA07 | Risedronic acid | Bisphosphonate |
| M05BA08 | Zoledronic acid | Bisphosphonate |
| N02AA01 | Morphine sulphate | Analgesics |
| N02AA03 | Hydromorphone | Analgesics |
| N02AA05 | Oxycodone | Analgesics |
| N02AA55 | Oxycodone + acetaminophen | Analgesics |
| N02AB03 | Fentanyl | Analgesics |
| N02AE01 | Buprenorphine | Analgesics |
| N02AX02 | Tramadol | Analgesics |
| N02AX06 | Tapentadol | Analgesics |
| N02BE01 | Paracetamol | Analgesics |
