# Supplementary material for: A direct method for the identification of patterns of care using administrative databases: the case of breast cancer
Source: Eur J Health Econ. 2021 Jul 26;22(9):1477–85. doi: 10.1007/s10198-021-01327-8 (PMC8558165; doi:10.1007/s10198-021-01327-8)
Supplement: Supplementary file 2 — Supplementary file2 (DOCX 30 kb) [file 10198_2021_1327_MOESM2_ESM.docx]

**European Journal of Health Economics**

**Title: A direct method for the identification of patterns of care using administrative databases: the case of breast cancer**

**Appendix 2**. List of 10 most frequent cancer-related codes in each database, by phase of care

| **Tab. A.2.1** - List of 10 most frequent cancer-related procedures/interventions in the hospital discharges database by phase of care: number of occurrences (N) and percentage over total occurrences (%) of cancer-related ICD9-CM codes | | | | |  |  |  |  |
| --- | --- | --- | --- | --- | --- | --- | --- | --- |
|  |  |  |  |  |  |  |  |  |
|  |  |  |  |  |  |  |  |  |
| Initial | N (%) |  | Continuing | N (%) | Final | N (%) |  |  |
| Resection of quadrant of breast (85.22) | *4719 (13.8)* |  | Personal history of malignant neoplasm of breast (V10.3) | *4015 (17.3)* | Injection or infusion of chemotherapeutic substance (99.25) | *1466 (14.9)* |  |  |
| Injection or infusion of chemotherapeutic substance (99.25) | *3905 (11.4)* |  | Injection or infusion of chemotherapeutic substance (99.25) | *2824 (12.2)* | Personal history of malignant neoplasm of breast (V10.3) | *1382 (14.1)* |  |  |
| Excision of axillary lymph node (40.23) | *3505 (10.3)* |  | Electrocardiogram (89.52) | *1418 (6.1)* | Routine chest x-ray (87.44) | *755 (7.7)* |  |  |
| Personal history of malignant neoplasm of breast (V10.3) | *2477 (7.3)* |  | Routine chest x-ray (87.44) | *1218 (5.2)* | Electrocardiogram (89.52) | *744 (7.6)* |  |  |
| Radical excision of axillary lymph nodes (40.51) | *1767 (5.2)* |  | Encounter for antineoplastic chemotherapy (V58.11) | *1099 (4.7)* | Encounter for antineoplastic chemotherapy (V58.11) | *667 (6.8)* |  |  |
| Encounter for antineoplastic chemotherapy (V58.11) | *1736 (5.1)* |  | Diagnostic ultrasound of heart (88.72) | *884 (3.8)* | Injection or infusion other therapeutic or prophylactic substance (99.29) | *628 (6.4)* |  |  |
| Scan of lymphatic system (92.16) | *1373 (4.0)* |  | Aftercare involving the use of plastic surgery (V51) | *720 (3.1)* | Computerized axial tomography of thorax (87.41) | *445 (4.5)* |  |  |
| Electrocardiogram (89.52) | *1186 (3.5)* |  | Injection or infusion other therapeutic or prophylactic substance (99.29) | *699 (3.0)* | Computerized axial tomography of head (87.03) | *380 (3.9)* |  |  |
| Routine chest x-ray (87.44) | *962 (2.8)* |  | Diagnostic ultrasound of abdomen and retroperitoneum (88.76) | *542 (2.3)* | Computerized axial tomography of abdomen (88.01) | *373 (3.8)* |  |  |
| Unilateral simple mastectomy (85.41) | *743 (2.2)* |  | Computerized axial tomography of head (87.03) | *535 (2.3)* | Diagnostic ultrasound of heart (88.72) | *373 (3.8)* |  |  |
| **Total** | **22,373 (65.5)** |  | **Total** | **13,954 (60.1)** | **Total** | ***7213 (73.4)*** |  |  |
|  |  |  |  | |  | |  |  |
|  |  |  |  | |  | |  |  |

| **Tab. A.2.2** List of 10 most frequent cancer-related procedures in the Outpatient service database by phase of care: number of occurrences (N) and percentage over total occurrences (%) of cancer-related ICD9-CM codes. | | | | | | | | | |  |  |  |
| --- | --- | --- | --- | --- | --- | --- | --- | --- | --- | --- | --- | --- |
|  | |  | |  |  | | | |  | | |  |
|  |  | | | | |  |  |  |  |  |  |  |
| Initial | | | N (%) |  | Continuing | | N (%) | Final | | | N (%) | |
| Check-up examination (89.01) | | | *12,733 (8.4)* |  | Check-up examination (89.01) | | *60,388 (14.7)* | Check-up examination (89.01) | | | *3909 (12.8)* | |
| First visit (89.7) | | | *11,045 (7.3)* |  | First visit (89.7) | | *50,755 (12.3)* | First visit (89.7) | | | *2946 (9.6)* | |
| Radiotherapy using LINAC (92.24.2) | | | *5367 (3.5)* |  | Bilateral mammography (87.37.1) | | *28,844 (7)* | Electrocardiogram (89.52) | | | *2016 (6.6)* | |
| Injection or infusion of chemotherapeutic substance (99.25) | | | *4778 (3.2)* |  | Electrocardiogram (89.52) | | *26,540 (6.4)* | Injection or infusion of chemotherapeutic substance (99.25) | | | *1598 (5.2)* | |
| Electrocardiogram (89.52) | | | *4722 (3.1)* |  | Bilateral ultrasound of breast (88.73.1) | | *26,504 (6.4)* | Routine x-ray of thorax (87.44.1) | | | *1111 (3.6)* | |
| Target acquisition via computerised tomography simulator (92.29.2) | | | *4420 (2.9)* |  | Ultrasound of whole abdomen (88.76.1) | | *21,239 (5.2)* | Computerized axial tomography (CAT) of thorax, without and with contrast (87.41.1) | | | *955 (3.1)* | |
| Bone or articular scintigraphy  (92.18.2) | | | *3969 (2.6)* |  | Routine x-ray of thorax (87.44.1) | | *20,427 (5)* | Computed tomography (CAT) of whole abdomen, with and without contrast (88.01.6) | | | *877 (2.9)* | |
| Other irrigation of wound (96.59) | | | *3912 (2.6)* |  | Transvaginal ultrasound (88.79.7) | | *9179 (2.2)* | Ultrasound of whole abdomen (88.76.1) | | | *577 (1.9)* | |
| Ultrasound of whole abdomen (88.76.1) | | | *3551 (2.3)* |  | Bone mineral density studies via Dual-energy X-ray absorptiometry (DXA) (88.99.3) | | *7682 (1.9)* | Other irrigation of wound (96.59) | | | *544 (1.8)* | |
| Bilateral ultrasound of breast (88.73.1) | | | *3500 (2.3)* |  | Mono-lateral mammography (87.37.2) | | *6708 (1.6)* | Injection of steroid or cortisone; subdermal implantation of progesterone or other hormone (99.23) | | | *526 (1.7)* | |
| **Total** | | | ***61,507 (40.6)*** |  | **Total** | | ***264,906 (64.4)*** | **Total** | | | ***15,585 (50.9)*** | |

|  |  |  |
| --- | --- | --- |
| **Tab. A.2.3** - List of 10 most frequent cancer-related drug prescriptions in the DP database by phase of care: number of occurrences (N) and percentage over total occurrences (%) of cancer-related ATC codes.   \| *Initial* \| N (%) \|  \| *Continuing* \| N (%) \|  \| *Final* \| N (%) \| \| --- \| --- \| --- \| --- \| --- \| --- \| --- \| --- \| \| Tamoxifen (L02BA01) \| *8078 (23.2)* \|  \| Tamoxifen (L02BA01) \| *36297 (22.8)* \|  \| Dexamethasone (H02AB02) \| *3713 (18)* \| \| Letrozole (L02BG04) \| *5794 (16.6)* \|  \| Anastrozole (L02BG03) \| *25072 (15.7)* \|  \| Fentanyl (N02AB03) \| *2377 (11.5)* \| \| Anastrozole (L02BG03) \| *4004 (11.5)* \|  \| Letrozole (L02BG04) \| *24245 (15.2)* \|  \| Prednisone (H02AB07) \| *1883 (9.1)* \| \| Prednisone (H02AB07) \| *1728 (5)* \|  \| Prednisone (H02AB07) \| *6667 (4.2)* \|  \| Morphine sulphate (N02AA01) \| *1271 (6.2)* \| \| Ondansetron (A04AA01) \| *1691 (4.9)* \|  \| Alendronic Acid (M05BA04) \| *5738 (3.6)* \|  \| Oxycodone (N02AA05) \| *1203 (5.8)* \| \| Dexamethasone (H02AB02) \| *1668 (4.8)* \|  \| Tramadol (N02AX02) \| *5509 (3.5)* \|  \| Tramadol (N02AX02) \| *908 (4.4)* \| \| Tramadol (N02AX02) \| *614 (1.8)* \|  \| Risedronic acid (M05BA07) \| *5221 (3.3)* \|  \| Anastrozole (L02BG03) \| *610 (3)* \| \| Betamethasone (H02AB01) \| *599 (1.7)* \|  \| Exemestane (L02BG06) \| *5129 (3.2)* \|  \| Betamethasone (H02AB01) \| *590 (2.9)* \| \| Lenograstim (L03AA10) \| *454 (1.3)* \|  \| Betamethasone (H02AB01) \| *4543 (2.9)* \|  \| Letrozole (L02BG04) \| *564 (2.7)* \| \| Granisetrone (A04AA02) \| *421 (1.2)* \|  \| Ibandronic acid (M05BA06) \| *3124 (2)* \|  \| Exemestane (L02BG06) \| *519 (2.5)* \| \| Total \| 25051 (71.9) \|  \| Total \| 121545 (76.4) \|  \| Total \| 13638 (66.2) \| | | |
|  |  |  |
