# Supplementary material for: A direct method for the identification of patterns of care using administrative databases: the case of breast cancer
Source: Eur J Health Econ. 2021 Jul 26;22(9):1477–85. doi: 10.1007/s10198-021-01327-8 (PMC8558165; doi:10.1007/s10198-021-01327-8)
Supplement: Supplementary file 3 — Supplementary file3 (DOCX 80 kb) [file 10198_2021_1327_MOESM3_ESM.docx]

**European Journal of Health Economics**

**Title: A direct method for the identification of patterns of care using administrative databases: the case of breast cancer**

**Appendix 3**. Occurrences and statistics of breast cancer-related codes listed in Appendix 1

**Tab. A.3.1** – Occurrences of cancer-related diagnoses and procedures in the Hospital Discharges (HD) database in the pre- and post-diagnosis for the subset of cases in initial phase of care. Column 1 illustrates the codes, expressed according to the ICD9-CM classification system; column 2 and 3 report the occurrences before and after cancer diagnosis, respectively; column 4 the results of the t-test; column 5 the corresponding probability.

**DIAGNOSTIC CODES**

| **ICD9-CM code** | **Pre-diagnosis occurrences** | **Post-diagnosis occurrences** | **t-test** | **prob** |
| --- | --- | --- | --- | --- |
| 174 | 0 | 0 | - | - |
| 174.0 | 5 | 77 | -7.955 | 1.81E-15 |
| 174.1 | 14 | 537 | -22.359 | 2.17E-110 |
| 174.2 | 26 | 607 | -23.184 | 1.62E-118 |
| 174.3 | 12 | 389 | -18.875 | 2.77E-79 |
| 174.4 | 95 | 2497 | -47.964 | 0.00E+00 |
| 174.5 | 16 | 446 | -20.064 | 2.55E-89 |
| 174.6 | 1 | 40 | -6.092 | 1.12E-09 |
| 174.8 | 36 | 1360 | -35.756 | 9.24E-278 |
| 174.9 | 69 | 2921 | -53.189 | 0.00E+00 |
| 196 | 0 | 0 | - | - |
| 196.0 | 1 | 15 | -3.500 | 4.65E-04 |
| 196.1 | 1 | 27 | -4.914 | 8.92E-07 |
| 196.2 | 13 | 19 | -1.061 | 2.89E-01 |
| 196.3 | 26 | 1889 | -43.117 | 0.00E+00 |
| 196.5 | 1 | 1 | 0.000 | 1.00E+00 |
| 196.6 | 3 | 0 | 1.732 | 8.33E-02 |
| 196.8 | 5 | 29 | -4.117 | 3.85E-05 |
| 196.9 | 1 | 46 | -6.566 | 5.20E-11 |
| 197 | 0 | 0 | - | - |
| 197.0 | 11 | 83 | -7.430 | 1.10E-13 |
| 197.1 | 0 | 2 | -1.414 | 1.57E-01 |
| 197.2 | 0 | 23 | -4.797 | 1.62E-06 |
| 197.3 | 0 | 0 | - | - |
| 197.4 | 0 | 1 | -1.000 | 3.17E-01 |
| 197.5 | 0 | 4 | -2.000 | 4.55E-02 |
| 197.6 | 3 | 40 | -5.644 | 1.67E-08 |
| 197.7 | 32 | 129 | -7.652 | 2.00E-14 |
| 197.8 | 3 | 7 | -1.265 | 2.06E-01 |
| 198 | 0 | 0 | - | - |
| 198.0 | 0 | 0 | - | - |
| 198.1 | 0 | 3 | -1.732 | 8.33E-02 |
| 198.2 | 2 | 18 | -3.578 | 3.46E-04 |
| 198.3 | 0 | 12 | -3.464 | 5.32E-04 |
| 198.4 | 0 | 0 | - | - |
| 198.5 | 7 | 182 | -12.744 | 3.64E-37 |
| 198.6 | 0 | 2 | -1.414 | 1.57E-01 |
| 198.7 | 3 | 2 | 0.447 | 6.55E-01 |
| 198.8 | 0 | 0 | - | - |
| 198.81 | 0 | 23 | -4.797 | 1.62E-06 |
| 198.82 | 0 | 0 | - | - |
| 198.89 | 0 | 14 | -3.742 | 1.83E-04 |
| 233.0 | 14 | 357 | -17.849 | 4.04E-71 |
| 238.3 | 11 | 66 | -6.270 | 3.62E-10 |
| 284.1 | 0 | 4 | -2.000 | 4.55E-02 |
| 288.03 | 1 | 43 | -6.334 | 2.41E-10 |
| 338.3 | 0 | 8 | -2.829 | 4.68E-03 |
| 528.01 | 0 | 6 | -2.450 | 1.43E-02 |
| 733.13 | 0 | 10 | -3.162 | 1.56E-03 |
| 733.15 | 0 | 4 | -2.000 | 4.55E-02 |
| 996.54 | 0 | 10 | -3.162 | 1.56E-03 |
| V10.3 | 14 | 2237 | -47.572 | 0.00E+00 |
| V43.82 | 0 | 13 | -3.606 | 3.11E-04 |
| V45.83 | 0 | 20 | -4.473 | 7.73E-06 |
| V50.41 | 0 | 6 | -2.450 | 1.43E-02 |
| V51 | 11 | 114 | -9.219 | 3.06E-20 |
| V52.4 | 1 | 190 | -13.693 | 1.26E-42 |
| V58.0 | 2 | 156 | -12.264 | 1.52E-34 |
| V58.1 | 0 | 341 | -18.509 | 2.61E-76 |
| V58.11 | 49 | 1623 | -38.908 | 0.00E+00 |
| V58.12 | 5 | 49 | -5.989 | 2.11E-09 |
| V58.81 | 11 | 190 | -12.641 | 1.36E-36 |
| V66.2 | 0 | 6 | -2.450 | 1.43E-02 |
| V86.0 | 8 | 329 | -17.524 | 1.28E-68 |
| V86.1 | 0 | 42 | -6.483 | 9.07E-11 |

**PROCEDURAL CODES**

| **ICD9-CM code** | **Pre-diagnosis occurrences** | **Post-diagnosis occurrences** | **t-test** | **prob** |
| --- | --- | --- | --- | --- |
| 33.26 | 1 | 9 | -2.530 | 1.14E-02 |
| 34.04 | 2 | 15 | -3.153 | 1.62E-03 |
| 34.91 | 2 | 11 | -2.496 | 1.26E-02 |
| 40.11 | 9 | 300 | -16.587 | 1.10E-61 |
| 40.19 | 0 | 191 | -13.838 | 1.70E-43 |
| 40.22 | 0 | 17 | -4.124 | 3.73E-05 |
| 40.23 | 70 | 3505 | -59.255 | 0.00E+00 |
| 40.29 | 18 | 392 | -18.517 | 2.15E-76 |
| 40.3 | 9 | 54 | -5.671 | 1.42E-08 |
| 40.5 | 0 | 0 | - | - |
| 40.50 | 0 | 11 | -3.317 | 9.11E-04 |
| 40.51 | 22 | 1764 | -41.712 | 0.00E+00 |
| 40.59 | 6 | 12 | -1.414 | 1.57E-01 |
| 50.11 | 2 | 6 | -1.414 | 1.57E-01 |
| 50.12 | 3 | 2 | 0.447 | 6.55E-01 |
| 50.19 | 0 | 2 | -1.414 | 1.57E-01 |
| 50.91 | 2 | 1 | 0.577 | 5.64E-01 |
| 54.24 | 0 | 2 | -1.414 | 1.57E-01 |
| 54.91 | 1 | 4 | -1.342 | 1.80E-01 |
| 68.12 | 8 | 65 | -6.675 | 2.49E-11 |
| 68.16 | 8 | 44 | -4.994 | 5.94E-07 |
| 85.0 | 0 | 10 | -3.162 | 1.56E-03 |
| 85.11 | 23 | 293 | -15.217 | 3.21E-52 |
| 85.12 | 13 | 113 | -8.917 | 4.92E-19 |
| 85.19 | 5 | 44 | -5.573 | 2.51E-08 |
| 85.20 | 8 | 146 | -11.131 | 9.32E-29 |
| 85.21 | 63 | 436 | -16.742 | 8.01E-63 |
| 85.22 | 116 | 4715 | -69.031 | 0.00E+00 |
| 85.23 | 1 | 12 | -3.051 | 2.28E-03 |
| 85.25 | 0 | 10 | -3.162 | 1.56E-03 |
| 85.33 | 0 | 81 | -9.005 | 2.21E-19 |
| 85.34 | 2 | 88 | -9.070 | 1.21E-19 |
| 85.35 | 0 | 14 | -3.742 | 1.83E-04 |
| 85.36 | 0 | 4 | -2.000 | 4.55E-02 |
| 85.41 | 8 | 739 | -26.878 | 2.17E-158 |
| 85.42 | 0 | 23 | -4.797 | 1.62E-06 |
| 85.43 | 1 | 432 | -20.773 | 1.42E-95 |
| 85.44 | 0 | 9 | -3.000 | 2.70E-03 |
| 85.45 | 7 | 568 | -23.484 | 1.61E-121 |
| 85.46 | 0 | 8 | -2.829 | 4.68E-03 |
| 85.47 | 0 | 33 | -5.746 | 9.18E-09 |
| 85.48 | 0 | 0 | - | - |
| 85.50 | 0 | 22 | -4.691 | 2.72E-06 |
| 85.51 | 0 | 1 | -1.000 | 3.17E-01 |
| 85.52 | 0 | 2 | -1.414 | 1.57E-01 |
| 85.53 | 0 | 239 | -15.485 | 5.36E-54 |
| 85.54 | 1 | 69 | -8.131 | 4.31E-16 |
| 85.6 | 2 | 108 | -10.114 | 4.96E-24 |
| 85.7 | 0 | 66 | -8.128 | 4.44E-16 |
| 85.81 | 0 | 32 | -5.658 | 1.54E-08 |
| 85.82 | 0 | 0 | - | - |
| 85.83 | 0 | 4 | -2.000 | 4.55E-02 |
| 85.84 | 0 | 20 | -4.473 | 7.73E-06 |
| 85.85 | 0 | 32 | -5.658 | 1.54E-08 |
| 85.86 | 1 | 36 | -5.755 | 8.68E-09 |
| 85.87 | 0 | 31 | -5.569 | 2.57E-08 |
| 85.89 | 2 | 384 | -19.493 | 2.02E-84 |
| 85.91 | 0 | 9 | -3.000 | 2.70E-03 |
| 85.93 | 0 | 9 | -3.000 | 2.70E-03 |
| 85.94 | 1 | 31 | -5.304 | 1.13E-07 |
| 85.95 | 7 | 641 | -25.013 | 1.60E-137 |
| 85.96 | 0 | 168 | -12.976 | 1.84E-38 |
| 85.99 | 0 | 91 | -9.545 | 1.40E-21 |
| 86.07 | 11 | 273 | -15.574 | 1.31E-54 |
| 86.11 | 1 | 10 | -2.714 | 6.65E-03 |
| 86.60 | 0 | 7 | -2.646 | 8.15E-03 |
| 87.03 | 22 | 131 | -8.821 | 1.16E-18 |
| 87.04 | 2 | 1 | 0.577 | 5.64E-01 |
| 87.17 | 0 | 4 | -2.000 | 4.55E-02 |
| 87.22 | 2 | 4 | -0.817 | 4.14E-01 |
| 87.23 | 1 | 6 | -1.890 | 5.88E-02 |
| 87.24 | 5 | 5 | 0.000 | 1.00E+00 |
| 87.29 | 2 | 4 | -0.817 | 4.14E-01 |
| 87.37 | 20 | 286 | -15.234 | 2.48E-52 |
| 87.38 | 0 | 0 | - | - |
| 87.41 | 34 | 362 | -16.520 | 3.27E-61 |
| 87.43 | 1 | 6 | -1.890 | 5.88E-02 |
| 87.44 | 121 | 955 | -25.574 | 9.46E-144 |
| 88.01 | 15 | 190 | -12.241 | 2.03E-34 |
| 88.02 | 3 | 7 | -1.265 | 2.06E-01 |
| 88.21 | 6 | 3 | 1.000 | 3.17E-01 |
| 88.26 | 9 | 16 | -1.400 | 1.61E-01 |
| 88.27 | 5 | 7 | -0.577 | 5.64E-01 |
| 88.31 | 0 | 9 | -3.000 | 2.70E-03 |
| 88.33 | 0 | 2 | -1.414 | 1.57E-01 |
| 88.38 | 6 | 56 | -6.352 | 2.13E-10 |
| 88.72 | 58 | 433 | -16.968 | 1.77E-64 |
| 88.73 | 17 | 280 | -15.288 | 1.08E-52 |
| 88.74 | 5 | 59 | -6.752 | 1.46E-11 |
| 88.75 | 4 | 2 | 0.817 | 4.14E-01 |
| 88.76 | 56 | 461 | -17.862 | 3.07E-71 |
| 88.79 | 9 | 176 | -12.292 | 1.07E-34 |
| 88.91 | 3 | 29 | -4.597 | 4.29E-06 |
| 88.92 | 3 | 41 | -5.730 | 1.01E-08 |
| 88.93 | 5 | 26 | -3.772 | 1.62E-04 |
| 88.94 | 1 | 4 | -1.342 | 1.80E-01 |
| 88.95 | 3 | 2 | 0.447 | 6.55E-01 |
| 88.97 | 4 | 19 | -3.128 | 1.76E-03 |
| 88.98 | 3 | 5 | -0.707 | 4.79E-01 |
| 89.04 | 1 | 11 | -2.887 | 3.89E-03 |
| 89.52 | 143 | 1170 | -28.546 | 1.86E-178 |
| 92.14 | 6 | 148 | -11.454 | 2.38E-30 |
| 92.16 | 36 | 1364 | -35.911 | 1.82E-279 |
| 92.18 | 1 | 40 | -6.092 | 1.12E-09 |
| 92.19 | 1 | 134 | -11.457 | 2.30E-30 |
| 92.2 | 0 | 0 | - | - |
| 92.20 | 0 | 9 | -3.000 | 2.70E-03 |
| 92.21 | 0 | 0 | - | - |
| 92.22 | 0 | 4 | -2.000 | 4.55E-02 |
| 92.23 | 0 | 3 | -1.732 | 8.33E-02 |
| 92.24 | 4 | 41 | -5.517 | 3.46E-08 |
| 92.25 | 1 | 135 | -11.501 | 1.39E-30 |
| 92.26 | 0 | 1 | -1.000 | 3.17E-01 |
| 92.27 | 0 | 5 | -2.236 | 2.53E-02 |
| 92.28 | 6 | 47 | -5.634 | 1.77E-08 |
| 92.29 | 1 | 196 | -13.911 | 6.12E-44 |
| 92.3 | 0 | 0 | - | - |
| 92.30 | 0 | 0 | - | - |
| 92.31 | 0 | 0 | - | - |
| 92.32 | 0 | 0 | - | - |
| 92.33 | 0 | 1 | -1.000 | 3.17E-01 |
| 92.39 | 0 | 0 | - | - |
| 99.0 | 0 | 4 | -2.000 | 4.55E-02 |
| 99.00 | 0 | 4 | -2.000 | 4.55E-02 |
| 99.02 | 0 | 0 | - | - |
| 99.03 | 9 | 30 | -3.363 | 7.70E-04 |
| 99.05 | 1 | 3 | -1.000 | 3.17E-01 |
| 99.1 | 1 | 1 | 0.000 | 1.00E+00 |
| 99.15 | 84 | 15 | 6.939 | <.0001 |
| 99.22 | 0 | 0 | - | - |
| 99.23 | 3 | 54 | -6.757 | 1.41E-11 |
| 99.24 | 2 | 6 | -1.414 | 1.57E-01 |
| 99.25 | 56 | 3473 | -58.891 | 0.00E+00 |
| 99.28 | 4 | 62 | -7.142 | 9.26E-13 |
| 99.29 | 62 | 413 | -16.145 | 1.47E-58 |
| 99.85 | 1 | 0 | 1.000 | 3.17E-01 |

**Tab. A.3.2** – Occurrences of cancer-related diagnoses and procedures in the Outpatient Services (OPS) database in the pre- and post-diagnosis for the subset of cases in initial phase of care. Column 1 illustrates the codes, expressed according to the ICD9-CM classification system; column 2 and 3 report the occurrences before and after cancer diagnosis, respectively; column 4 the results of the t-test; column 5 the corresponding probability.

| **ICD9-CM code** | **Pre-diagnosis occurrences** | **Post-diagnosis occurrences** | **t-test** | **prob** |
| --- | --- | --- | --- | --- |
| 03.8 | 0 | 0 | - | - |
| 33.26 | 0 | 0 | - | - |
| 34.91 | 0 | 2 | -1.414 | 1.57E-01 |
| 34.91.1 | 0 | 0 | - | - |
| 38.93.2 | 0 | 1 | -1.000 | 3.17E-01 |
| 38.99.1 | 1 | 15 | -3.500 | 4.65E-04 |
| 38.99.2 | 0 | 0 | - | - |
| 40.11 | 4 | 3 | 0.378 | 7.05E-01 |
| 40.19.1 | 6 | 161 | -12.011 | 3.41E-33 |
| 40.19.2 | 0 | 0 | - | - |
| 50.11 | 1 | 2 | -0.577 | 5.64E-01 |
| 50.19.1 | 0 | 0 | - | - |
| 50.91 | 0 | 0 | - | - |
| 50.91.1 | 0 | 0 | - | - |
| 54.24 | 0 | 0 | - | - |
| 54.24.1 | 0 | 1 | -1.000 | 3.17E-01 |
| 54.91 | 0 | 0 | - | - |
| 54.91.1 | 0 | 0 | - | - |
| 68.12.1 | 42 | 69 | -9.040 | 1.59E-19 |
| 68.16.1 | 10 | 24 | -2.401 | 1.63E-02 |
| 83.21 | 2 | 0 | 1.414 | 1.57E-01 |
| 83.21.1 | 8 | 13 | -1.091 | 2.75E-01 |
| 85.0 | 0 | 0 | - | - |
| 85.11 | 164 | 201 | -1.940 | 5.24E-02 |
| 85.11.1 | 1066 | 1157 | -1.949 | 5.13E-02 |
| 85.2 | 0 | 0 | - | - |
| 85.20 | 3 | 1 | 1.000 | 3.17E-01 |
| 85.21 | 2 | 8 | -1.897 | 5.78E-02 |
| 85.21.1 | 24 | 27 | -0.420 | 6.74E-01 |
| 86.01 | 0 | 17 | -4.124 | 3.73E-05 |
| 86.01.1 | 0 | 139 | -11.804 | 4.05E-32 |
| 86.07 | 0 | 34 | -5.833 | 5.48E-09 |
| 86.11 | 14 | 16 | -0.365 | 7.15E-01 |
| 86.4 | 3 | 77 | -8.279 | 1.27E-16 |
| 86.60 | 0 | 0 | - | - |
| 87.03 | 9 | 138 | -10.652 | 1.80E-26 |
| 87.03.1 | 42 | 274 | -13.079 | 4.81E-39 |
| 87.03.7 | 4 | 26 | -4.017 | 5.89E-05 |
| 87.03.8 | 3 | 102 | -9.670 | 4.20E-22 |
| 87.17.1 | 3 | 99 | -9.513 | 1.91E-21 |
| 87.17.4 | 0 | 0 | - | - |
| 87.22 | 14 | 192 | -12.421 | 2.19E-35 |
| 87.23 | 11 | 257 | -15.059 | 3.69E-51 |
| 87.24 | 23 | 362 | -17.329 | 4.05E-67 |
| 87.24.1 | 5 | 83 | -8.321 | 8.93E-17 |
| 87.24.2 | 0 | 4 | -2.000 | 4.55E-02 |
| 87.29 | 15 | 20 | -0.845 | 3.98E-01 |
| 87.35 | 4 | 3 | 0.000 | 0.00E+00 |
| 87.37.1 | 1230 | 1325 | -1.901 | 5.73E-02 |
| 87.37.2 | 40 | 566 | -21.467 | 7.13E-102 |
| 87.41 | 5 | 132 | -10.862 | 1.84E-27 |
| 87.41.1 | 28 | 924 | -29.270 | 5.05E-187 |
| 87.42.1 | 0 | 0 | - | - |
| 87.42.2 | 0 | 0 | - | - |
| 87.42.3 | 0 | 1 | -1.000 | 3.17E-01 |
| 87.43.1 | 4 | 26 | -4.017 | 5.89E-05 |
| 87.43.2 | 5 | 201 | -13.679 | 1.56E-42 |
| 87.44.1 | 68 | 1932 | -42.377 | 0.00E+00 |
| 88.01.1 | 2 | 12 | -2.673 | 7.52E-03 |
| 88.01.2 | 15 | 86 | -7.069 | 1.57E-12 |
| 88.01.3 | 2 | 13 | -2.840 | 4.51E-03 |
| 88.01.4 | 9 | 36 | -4.026 | 5.68E-05 |
| 88.01.5 | 19 | 59 | -4.531 | 5.88E-06 |
| 88.01.6 | 31 | 887 | -28.466 | 4.39E-177 |
| 88.21 | 13 | 228 | -13.876 | 1.03E-43 |
| 88.26 | 27 | 325 | -15.925 | 5.37E-57 |
| 88.27 | 16 | 229 | -13.634 | 2.89E-42 |
| 88.27.3 | 0 | 5 | -2.236 | 2.53E-02 |
| 88.31 | 2 | 10 | -2.310 | 2.09E-02 |
| 88.33.2 | 2 | 1 | 0.577 | 5.64E-01 |
| 88.38.1 | 5 | 102 | -9.385 | 6.47E-21 |
| 88.38.2 | 0 | 6 | -1.604 | 1.09E-01 |
| 88.38.3 | 1 | 5 | -1.886 | 5.93E-02 |
| 88.38.4 | 1 | 0 | -1.000 | 3.17E-01 |
| 88.38.5 | 4 | 38 | -5.248 | 1.54E-07 |
| 88.38.6 | 8 | 5 | -0.707 | 4.79E-01 |
| 88.38.7 | 0 | 5 | -2.236 | 2.53E-02 |
| 88.72.1 | 25 | 277 | -14.533 | 8.78E-48 |
| 88.73.1 | 1938 | 1957 | -0.310 | 7.57E-01 |
| 88.73.2 | 63 | 833 | -25.900 | 3.59E-147 |
| 88.73.4 | 2 | 60 | -7.370 | 1.73E-13 |
| 88.73.6 | 59 | 63 | -0.362 | 7.17E-01 |
| 88.73.7 | 12 | 192 | -12.623 | 1.75E-36 |
| 88.74.1 | 192 | 1399 | -30.597 | 2.67E-204 |
| 88.75.1 | 3 | 107 | -9.925 | 3.37E-23 |
| 88.76.1 | 67 | 2573 | -49.877 | 0.00E+00 |
| 88.78.2 | 10 | 93 | -8.184 | 2.78E-16 |
| 88.79.7 | 196 | 1357 | -29.777 | 1.09E-193 |
| 88.90.2 | 16 | 1079 | -32.426 | 1.22E-228 |
| 88.90.3 | 21 | 32 | -1.511 | 1.31E-01 |
| 88.91.1 | 44 | 47 | -0.315 | 7.53E-01 |
| 88.91.2 | 3 | 79 | -8.398 | 4.62E-17 |
| 88.92 | 1 | 3 | -1.000 | 3.17E-01 |
| 88.92.1 | 2 | 6 | -1.414 | 1.57E-01 |
| 88.92.6 | 4 | 6 | -0.632 | 5.27E-01 |
| 88.92.7 | 18 | 18 | 0.000 | 1.00E+00 |
| 88.92.8 | 13 | 32 | -2.833 | 4.61E-03 |
| 88.92.9 | 22 | 514 | -21.343 | 1.03E-100 |
| 88.93 | 12 | 156 | -11.124 | 1.02E-28 |
| 88.93.1 | 6 | 29 | -3.889 | 1.01E-04 |
| 88.94.1 | 50 | 66 | -1.486 | 1.37E-01 |
| 88.94.2 | 7 | 8 | -1.808 | 7.07E-02 |
| 88.95.1 | 6 | 10 | -1.000 | 3.17E-01 |
| 88.95.2 | 3 | 66 | -7.588 | 3.28E-14 |
| 88.95.3 | 0 | 0 | - | - |
| 88.95.4 | 5 | 8 | -0.832 | 4.05E-01 |
| 88.95.5 | 30 | 37 | -0.855 | 3.92E-01 |
| 88.95.6 | 1 | 0 | 1.000 | 3.17E-01 |
| 88.99.1 | 0 | 0 | - | - |
| 88.99.2 | 176 | 1127 | -26.578 | 6.36E-155 |
| 88.99.3 | 2 | 55 | -7.023 | 2.19E-12 |
| 88.99.4 | 1 | 7 | -2.121 | 3.39E-02 |
| 88.99.5 | 5 | 12 | -1.698 | 8.95E-02 |
| 89.01 | 1923 | 11295 | -89.655 | 0.00E+00 |
| 89.03 | 25 | 2028 | -45.000 | 0.00E+00 |
| 89.07 | 32 | 419 | -18.286 | 1.64E-74 |
| 89.52 | 95 | 1802 | -39.793 | 0.00E+00 |
| 89.7 | 328 | 9338 | -99.849 | 0.00E+00 |
| 90.04.5 | 363 | 13031 | -124.049 | 0.00E+00 |
| 90.05.1 | 97 | 1208 | -31.061 | 2.59E-210 |
| 90.05.5 | 203 | 234 | -1.486 | 1.37E-01 |
| 90.07.5 | 9 | 11 | -0.447 | 6.55E-01 |
| 90.09.2 | 353 | 12661 | -121.780 | 0.00E+00 |
| 90.10.4 | 527 | 4602 | -59.098 | 0.00E+00 |
| 90.10.5 | 1168 | 6060 | -60.384 | 0.00E+00 |
| 90.11.4 | 1078 | 7164 | -71.104 | 0.00E+00 |
| 90.13.3 | 58 | 2009 | -43.664 | 0.00E+00 |
| 90.15.4 | 74 | 836 | -25.432 | 5.18E-142 |
| 90.16.3 | 388 | 13404 | -126.100 | 0.00E+00 |
| 90.16.4 | 12 | 167 | -11.601 | 4.36E-31 |
| 90.19.2 | 101 | 534 | -17.253 | 1.41E-66 |
| 90.23.3 | 13 | 528 | -22.242 | 3.73E-109 |
| 90.23.5 | 1326 | 8569 | -78.180 | 0.00E+00 |
| 90.24.1 | 4 | 94 | -9.099 | 9.42E-20 |
| 90.25.5 | 227 | 8921 | -98.704 | 0.00E+00 |
| 90.27.1 | 372 | 9807 | -102.347 | 0.00E+00 |
| 90.29.2 | 727 | 5023 | -59.007 | 0.00E+00 |
| 90.30.4 | 0 | 1 | -1.000 | 3.17E-01 |
| 90.32.3 | 10 | 473 | -21.153 | 5.99E-99 |
| 90.37.4 | 223 | 7670 | -89.876 | 0.00E+00 |
| 90.38.1 | 5 | 190 | -13.270 | 3.96E-40 |
| 90.38.4 | 154 | 3154 | -53.588 | 0.00E+00 |
| 90.38.5 | 242 | 1698 | -33.503 | 3.02E-244 |
| 90.39.5 | 50 | 317 | -13.972 | 2.64E-44 |
| 90.40.1 | 48 | 313 | -13.981 | 2.31E-44 |
| 90.40.4 | 193 | 7131 | -86.482 | 0.00E+00 |
| 90.44.1 | 226 | 8026 | -92.393 | 0.00E+00 |
| 90.55.1 | 30 | 1087 | -31.923 | 9.12E-222 |
| 90.55.2 | 363 | 5340 | -68.998 | 0.00E+00 |
| 90.55.3 | 82 | 1239 | -32.163 | 3.49E-225 |
| 90.55.4 | 1 | 1 | 0.000 | 1.00E+00 |
| 90.55.5 | 0 | 0 | - | - |
| 90.56.1 | 1 | 3 | -1.000 | 3.17E-01 |
| 90.56.2 | 0 | 0 | - | - |
| 90.56.3 | 106 | 5170 | -73.021 | 0.00E+00 |
| 90.56.4 | 4 | 15 | -2.524 | 1.16E-02 |
| 90.62.2 | 491 | 19221 | -162.647 | 0.00E+00 |
| 90.70.4 | 32 | 294 | -14.545 | 7.39E-48 |
| 90.70.5 | 2 | 1 | 0.577 | 5.64E-01 |
| 90.71.3 | 6 | 37 | -4.729 | 2.26E-06 |
| 90.71.31 | 6 | 2 | 1.414 | 1.57E-01 |
| 90.71.32 | 0 | 2 | -1.414 | 1.57E-01 |
| 90.75.4 | 176 | 3068 | -52.117 | 0.00E+00 |
| 90.76.1 | 74 | 1359 | -34.335 | 5.26E-256 |
| 90.94.1 | 1 | 7 | -2.121 | 3.39E-02 |
| 91.29.1 | 0 | 0 | - | - |
| 91.29.2 | 0 | 4 | -2.000 | 4.55E-02 |
| 91.29.3 | 11 | 22 | -1.915 | 5.55E-02 |
| 91.29.4 | 15 | 32 | -2.480 | 1.31E-02 |
| 91.29.5 | 0 | 0 | - | - |
| 91.30.1 | 2 | 4 | -0.817 | 4.14E-01 |
| 91.36.1 | 18 | 27 | -1.342 | 1.80E-01 |
| 91.36.3 | 1 | 2 | -0.577 | 5.64E-01 |
| 91.36.5 | 7 | 154 | -11.600 | 4.43E-31 |
| 91.37.1 | 3 | 19 | -3.412 | 6.46E-04 |
| 91.37.2 | 1 | 13 | -3.207 | 1.34E-03 |
| 91.37.3 | 17 | 364 | -17.832 | 6.01E-71 |
| 91.37.4 | 6 | 190 | -13.164 | 1.60E-39 |
| 91.37.5 | 0 | 0 | - | - |
| 91.38.4 | 0 | 18 | -4.243 | 2.21E-05 |
| 91.39.1 | 861 | 942 | -1.923 | 5.45E-02 |
| 91.39.3 | 6 | 7 | -0.277 | 7.81E-01 |
| 91.39.6 | 21 | 220 | -12.841 | 1.06E-37 |
| 91.40.4 | 7 | 5 | 0.577 | 5.64E-01 |
| 91.40.5 | 53 | 74 | -1.865 | 6.23E-02 |
| 91.41.1 | 50 | 54 | -0.392 | 6.95E-01 |
| 91.41.2 | 1 | 1 | 0.000 | 1.00E+00 |
| 91.44.3 | 3 | 6 | -1.000 | 3.17E-01 |
| 91.44.5 | 7 | 25 | -3.183 | 1.46E-03 |
| 91.46.5 | 59 | 1109 | -31.010 | 1.45E-209 |
| 91.47.1 | 75 | 100 | -1.891 | 5.86E-02 |
| 91.47.2 | 6 | 19 | -2.600 | 9.31E-03 |
| 91.47.3 | 0 | 1 | -1.000 | 3.17E-01 |
| 91.47.4 | 1 | 7 | -2.121 | 3.39E-02 |
| 91.48.4 | 38 | 436 | -18.346 | 5.46E-75 |
| 91.49.2 | 707 | 21828 | -177.166 | 0.00E+00 |
| 92.11.5 | 0 | 1 | -1.000 | 3.17E-01 |
| 92.11.6 | 0 | 1 | -1.000 | 3.17E-01 |
| 92.14.1 | 1 | 21 | -4.265 | 2.00E-05 |
| 92.14.2 | 4 | 19 | -3.128 | 1.76E-03 |
| 92.16.1 | 26 | 130 | -8.335 | 7.88E-17 |
| 92.18.1 | 1 | 1 | 0.000 | 1.00E+00 |
| 92.18.2 | 125 | 2956 | -52.316 | 0.00E+00 |
| 92.18.4 | 0 | 0 | - | - |
| 92.18.5 | 1 | 1 | 0.000 | 1.00E+00 |
| 92.18.6 | 12 | 410 | -19.442 | 6.13E-84 |
| 92.19.6 | 9 | 278 | -15.916 | 6.39E-57 |
| 92.19.8 | 4 | 57 | -6.789 | 1.14E-11 |
| 92.23.1 | 0 | 8 | -2.829 | 4.68E-03 |
| 92.23.2 | 0 | 2 | -1.414 | 1.57E-01 |
| 92.23.3 | 0 | 0 | - | - |
| 92.24.1 | 3 | 881 | -29.759 | 3.84E-193 |
| 92.24.2 | 0 | 3393 | -60.054 | 0.00E+00 |
| 92.24.3 | 0 | 35 | -5.918 | 3.28E-09 |
| 92.24.4 | 20 | 2717 | -52.810 | <.0001 |
| 92.25.1 | 6 | 1409 | -37.763 | 2.71E-308 |
| 92.28.4 | 0 | 0 | - | - |
| 92.28.5 | 0 | 0 | - | - |
| 92.28.6 | 0 | 1 | -1.000 | 3.17E-01 |
| 92.29.1 | 7 | 2046 | -45.824 | 0.00E+00 |
| 92.29.2 | 9 | 2889 | -54.896 | 0.00E+00 |
| 92.29.3 | 0 | 0 | - | - |
| 92.29.4 | 5 | 1158 | -34.155 | 3.80E-253 |
| 92.29.5 | 1 | 1257 | -35.806 | 1.03E-277 |
| 92.29.6 | 2 | 1244 | -35.573 | 3.54E-274 |
| 92.29.7 | 7 | 1430 | -38.013 | 0.00E+00 |
| 92.29.8 | 3 | 1332 | -36.803 | 4.31E-293 |
| 93.01.1 | 17 | 169 | -11.160 | 6.75E-29 |
| 93.01.2 | 7 | 18 | -2.200 | 2.78E-02 |
| 93.04.1 | 1 | 15 | -3.500 | 4.65E-04 |
| 93.04.2 | 18 | 106 | -7.909 | 2.63E-15 |
| 93.11.1 | 2 | 46 | -6.353 | 2.12E-10 |
| 93.11.2 | 15 | 132 | -9.660 | 4.60E-22 |
| 93.11.3 | 21 | 108 | -7.666 | 1.79E-14 |
| 93.11.4 | 66 | 377 | -14.819 | 1.30E-49 |
| 93.11.5 | 2 | 90 | -9.182 | 4.37E-20 |
| 93.12.2 | 0 | 0 | - | - |
| 93.39.2 | 0 | 207 | -14.414 | 5.14E-47 |
| 93.40.2 | 0 | 0 | - | - |
| 94.09 | 81 | 554 | -18.852 | 4.36E-79 |
| 94.3 | 3 | 49 | -6.382 | 1.76E-10 |
| 94.32 | 0 | 0 | - | - |
| 94.42 | 12 | 23 | -1.860 | 6.29E-02 |
| 94.44 | 6 | 128 | -10.550 | 5.31E-26 |
| 96.59 | 281 | 3374 | -52.621 | 0.00E+00 |
| 99.07.1 | 3 | 4 | -0.707 | 4.79E-01 |
| 99.23 | 78 | 2305 | -46.536 | 0.00E+00 |
| 99.24.1 | 0 | 103 | -10.158 | 3.20E-24 |
| 99.25 | 138 | 4507 | -66.703 | 0.00E+00 |
| 99.85 | 0 | 7 | -2.646 | 8.15E-03 |

**Tab. A.3.3** – Occurrences of cancer-related drugs in the Drug Prescription (DP) database in the pre- and post-diagnosis for the subset of cases in initial phase of care. Column 1 illustrates the codes, expressed according to the ATC classification system; column 2 and 3 report the occurrences before and after cancer diagnosis, respectively; column 4 the results of the t-test; column 5 the corresponding probability.

| **ATC code** | **Pre-diagnosis occurrences** | **Post-diagnosis occurrences** | **t-test** | **prob** |
| --- | --- | --- | --- | --- |
| A03FA | 3 | 12 | -2.324 | 2.01E-02 |
| A03FA01 | 32 | 118 | -7.028 | 2.11E-12 |
| A03FA05 | 1 | 70 | -8.194 | 2.58E-16 |
| A04AA01 | 109 | 1691 | -37.818 | 0.00E+00 |
| A04AA02 | 17 | 506 | -21.474 | 6.42E-102 |
| A04AA03 | 18 | 102 | -7.674 | 1.69E-14 |
| A04AA05 | 6 | 180 | -12.778 | 2.44E-37 |
| A04AD12 | 4 | 161 | -12.239 | 2.11E-34 |
| A07DA03 | 0 | 4 | -2.000 | 4.55E-02 |
| B03XA01 | 2 | 12 | -2.673 | 7.52E-03 |
| B03XA02 | 1 | 15 | -3.500 | 4.65E-04 |
| H02AB01 | 253 | 445 | -7.291 | 3.09E-13 |
| H02AB02 | 108 | 1668 | -37.536 | 0.00E+00 |
| H02AB04 | 32 | 103 | -6.115 | 9.68E-10 |
| H02AB07 | 374 | 1728 | -29.923 | 1.13E-195 |
| H02AB09 | 25 | 99 | -6.650 | 2.94E-11 |
| H02AB10 | 18 | 24 | -0.926 | 3.54E-01 |
| L01AA01 | 13 | 731 | -26.490 | 1.00E-153 |
| L01BA01 | 83 | 2095 | -43.892 | 0.00E+00 |
| L01BC02 | 122 | 1679 | -37.203 | 8.08E-300 |
| L01BC05 | 0 | 12 | -3.464 | 5.32E-04 |
| L01BC06 | 42 | 175 | -9.040 | 1.59E-19 |
| L01CA04 | 0 | 23 | -4.797 | 1.62E-06 |
| L01CD01 | 103 | 2481 | -47.786 | 0.00E+00 |
| L01CD02 | 5 | 669 | -25.726 | 3.92E-145 |
| L01DB01 | 29 | 797 | -26.903 | 1.66E-158 |
| L01DB02 | 0 | 0 | - | - |
| L01DB03 | 4 | 387 | -19.434 | 7.30E-84 |
| L01XC03 | 5 | 31 | -4.334 | 1.46E-05 |
| L01XC07 | 0 | 0 | - | - |
| L01XC14 | 0 | 0 | - | - |
| L01XE07 | 0 | 0 | - | - |
| L02AB01 | 1 | 9 | -2.530 | 1.14E-02 |
| L02AE01 | 0 | 0 | - | - |
| L02AE02 | 5 | 767 | -27.609 | 1.04E-166 |
| L02AE03 | 0 | 91 | -9.547 | 1.39E-21 |
| L02AE04 | 4 | 669 | -25.784 | 8.95E-146 |
| L02BA01 | 49 | 6634 | -85.621 | 0.00E+00 |
| L02BA02 | 0 | 4 | -2.000 | 4.55E-02 |
| L02BA03 | 0 | 7 | -2.646 | 8.15E-03 |
| L02BG03 | 13 | 3523 | -60.922 | 0.00E+00 |
| L02BG04 | 29 | 5191 | -74.900 | 0.00E+00 |
| L02BG06 | 9 | 451 | -20.688 | 9.51E-95 |
| L03AA02 | 8 | 213 | -13.815 | 2.42E-43 |
| L03AA10 | 15 | 454 | -20.349 | 9.48E-92 |
| L03AA13 | 5 | 608 | -24.484 | 1.01E-131 |
| M05BA | 33 | 52 | -2.062 | 3.92E-02 |
| M05BA04 | 234 | 302 | -2.944 | 3.24E-03 |
| M05BA06 | 91 | 156 | -4.141 | 3.47E-05 |
| M05BA07 | 127 | 192 | -3.645 | 2.68E-04 |
| M05BA08 | 9 | 193 | -12.967 | 2.11E-38 |
| N02AA01 | 13 | 130 | -9.794 | 1.23E-22 |
| N02AA03 | 2 | 37 | -5.606 | 2.08E-08 |
| N02AA05 | 47 | 183 | -8.980 | 2.77E-19 |
| N02AA55 | 17 | 92 | -7.189 | 6.60E-13 |
| N02AB03 | 45 | 203 | -10.048 | 9.65E-24 |
| N02AE01 | 9 | 42 | -4.622 | 3.80E-06 |
| N02AX02 | 336 | 470 | -4.737 | 2.17E-06 |
| N02AX06 | 8 | 34 | -4.013 | 6.00E-05 |
| N02BE01 | 1 | 5 | -1.633 | 1.02E-01 |
